# Schiff Base Compounds Derived from 5-Methyl Salicylaldehyde as Turn-On Fluorescent Probes for Al^3+^ Detection: Experimental and DFT Calculations

**DOI:** 10.3390/molecules30051128

**Published:** 2025-02-28

**Authors:** Huan-Qing Li, Shi-Hang Yang, Yun Li, Wan-Xin Ye, Zi-Yu Liao, Jia-Qian Lu, Zhao-Yang Wang

**Affiliations:** School of Chemistry, South China Normal University, Guangzhou Key Laboratory of Analytical Chemistry for Biomedicine, GDMPA Key Laboratory for Process Control and Quality Evaluation of Chiral Pharmaceuticals, Key Laboratory of Theoretical Chemistry of Environment, Ministry of Education, Guangzhou 510006, China; 2022022611@m.scnu.edu.cn (H.-Q.L.); 2024022743@m.scnu.edu.cn (S.-H.Y.); 2024022879@m.scnu.edu.cn (Y.L.); 2024022841@m.scnu.edu.cn (W.-X.Y.); 20212421083@m.scnu.edu.cn (Z.-Y.L.); 20222421008@m.scnu.edu.cn (J.-Q.L.)

**Keywords:** Schiff base, 5-methyl salicylaldehyde, Al^3+^ fluorescent probe, excited state intramolecular proton transfer, chelation-enhanced fluorescence mechanism, DFT calculations

## Abstract

Using 5-methyl salicylaldehyde (**2**) as a reactant to react with different amines, 2-aminobenzimidazole (**1a**), 2-aminobenzothiazole (**1b**), and 2-aminopyridine (**1c**), respectively, three types of Schiff base fluorescent probes **3a**–**3c** were designed and synthesized for selective detection of Al^3+^ in aqueous media. The structure of the compounds was acquired by ^1^H NMR, ^13^C NMR, and X-ray single-crystal diffraction. Furthermore, their photochromic and fluorescent behaviors have been investigated systematically by fluorescence spectra. Compounds **3a**–**3c** can exhibit high selectivity, sensitivity, and anti-interference properties towards Al^3+^ in aqueous media. Among them, the limit of detection (LOD) of probe **3b** for Al^3+^ is 2.81 × 10^−7^ M. Notably, the response times of probes **3a**–**3c** for Al^3+^ are 90 s, 80 s, and 80 s, respectively, which are much faster than most previously reported probes. The coordination stoichiometry between compounds **3a**–**3c** and Al^3+^ has been verified to be 1:1 through the Job’s plot. After coordination with Al^3+^, the C=N isomerization of compounds **3a**–**3c** is inhibited, leading to the closure of the excited state intramolecular proton transfer (ESIPT) effect. At the same time, the fluorescence intensity is significantly increased through chelation-enhanced fluorescence mechanism (CHEF), which is confirmed by density functional theory (DFT) calculations. In addition, probes **3a**–**3c** can be potentially applied in the selective and high-precision detection of Al^3+^ in environmental systems.

## 1. Introduction

It is well known that aluminum is the most abundant metal element in the Earth’s crust [1] and is widely used in many fields, such as food packaging, building materials, and electronic devices [2]. Due to the widespread presence of aluminum products in people’s daily lives, the harm of aluminum to humans has attracted widespread attention in recent years [3]. Al^3+^ is a neurotoxin and a “silent killer” in the human body [4]. The World Health Organization (WHO) has listed Al^3+^ as a source of food contamination and set the maximum allowable concentration in drinking water at 7.41 μM [5]. Long-term excessive intake of Al^3+^ can lead to major diseases such as Parkinson’s disease, Alzheimer’s disease, and rickets [6]. In addition, high concentrations of Al^3+^ deposition can lead to soil acidification, which can seriously harm crops and soil ecosystems [7]. Therefore, developing a selective and effective method for detecting Al^3+^ is crucial for human health and ecological protection.

Traditional Al^3+^ analysis methods, such as inductively coupled plasma optical emission spectroscopy (ICP OES) [8], atomic absorption spectroscopy (AAS) [9], and capillary electrophoresis (CE) [10], can accurately determine the content of Al^3+^. However, these detection techniques still have many drawbacks, such as complex processes, high detection costs, the need for very professional personnel, and the need for a large number of sample pretreatment [11,12].

In recent years, fluorescence probe detection technology has become an effective method for detecting Al^3+^ due to its simple operation, good selectivity, and real-time detection [13], which has attracted widespread attention from researchers [14,15]. Due to the strong hydration ability of Al^3+^, its coordination with transition metal ions is relatively poor and lacks spectral characteristics. Therefore, many research groups have recently made new breakthroughs in the development of fluorescent probes to address this issue [16,17,18]. However, some fluorescent probes still have various other problems, including long response times, high detection limits, and poor anti-interference ability [19,20,21]. Therefore, fluorescence technology for detecting Al^3+^ still poses significant challenges [22].

Considering the Hard Soft Acid Base Theory (HSAB), Al^3+^ is a hard acid that tends to bind to coordination molecules with donor atoms such as N and O to form stable complexes [23,24]. Therefore, most reported Al^3+^ probes contain hard base donor sites, such as N and O atoms [25,26,27]. Among them, Schiff base compounds are widely used in the development of Al^3+^ fluorescent probes due to their advantages, such as simple synthesis routes, strong metal ion coordination ability, and fast response speed [28,29,30]. However, the structure-activity relationship of the obtained Schiff base Al^3+^ fluorescent probes based on different nitrogen heterocyclic compounds still needs to be deeply studied, especially the influence of different nitrogen heterocyclic fluorescent groups needs to be explored.

**Scheme 1:**
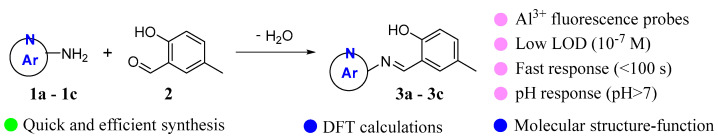
Design of Schiff base probes **3a**–**3c** with nitrogen-containing heterocyclic structure for Al^3+^ detection.

In view of this, based on our previous research on benzazole-based metal ion fluorescent probes [31,32], herein, we further designed three fluorescent probes containing benzimidazole group, benzothiazole group, and pyridine group (Scheme 1). Three Schiff base fluorescent probes **3a**–**3c** were rapidly, efficiently, and environmentally synthesized through a simple one-step Schiff base condensation reaction by using 2-aminobenzimidazole (**1a**), 2-aminobenzothiazole (**1b**), 2-aminopyridine (**1c**), and 5-methyl salicylaldehyde (**2**) as starting materials.

The test results indicate that they can selectively detect Al^3+^ with a low detection limit and fast detection speed. Furthermore, we systematically studied the relationship between the structure and properties of fluorescent groups in Schiff base probes with different nitrogen heterocyclic structures using density functional theory (DFT) calculations, which can provide the guidance for the design and synthesis of novel Schiff base Al^3+^ fluorescent probes.

## 2. Results and Discussion

### 2.1. Design, Synthesis, and Structural Characterization of Compounds ***3a**–**3c***

Salicylaldehyde and its derivatives are commonly found bioactive elements in cosmetics and natural products [33]. They can also be used as raw materials to participate in the construction of conjugated metal–organic frameworks (c-MOF) materials [34], photochromic materials [35], nanomaterials [36], and fluorescent probes [37]. Primarily, due to their excellent photostability and ability to regulate fluorescence properties [38], salicylaldehyde and its derivatives have been widely used as fluorescent probes for detecting cations [39], anions [40], and monitoring pH changes in biological systems [41].

By using the reaction of the aldehyde group, salicylaldehyde often reacts with heterocyclic amine compounds to synthesize Schiff base probe molecules through a one-step Schiff base reaction [39,42]. In addition, the hydroxyl group of salicylaldehyde can also participate in coordination as a proton donor to detect metal ions [43]. The optical properties of Schiff base compounds can be easily adjusted by incorporating different fluorescent groups into their molecular skeleton [25], while *N*-heterocyclic compounds have good fluorescence properties and are often used to synthesize organic small molecule fluorescent probes [44,45]. Therefore, we designed three Schiff base fluorescent probes based on 5-methyl salicylaldehyde by introducing different nitrogen heterocyclic fluorescent groups, respectively.

**Scheme 2:**
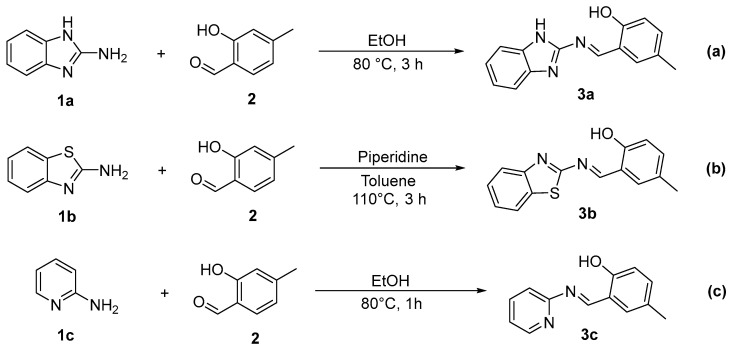
(**a**–**c**) Synthetic route of compounds **3a**–**3c**.

At present, most Schiff base probe molecules are synthesized using anhydrous ethanol [46] or methanol [47] as solvent. In the synthesis process of compounds **3a**–**3c**, we found that both compounds **3a** and **3c** can be synthesized by the above method with satisfactory yields. However, when compound **3b** is synthesized using anhydrous ethanol as a solvent, the yield is relatively low. The reason for this may be related to the molecular structure of substrates **1a**–**1c** (Scheme 2).

The imidazole structure in **1a** and the pyridine structure in **1c** make the entire molecule more alkaline, except that the substrates all have primary amino groups due to the electron-withdrawing property of benzothiazole, and **1b** can stabilize the conjugated system of the entire molecule, making the alkalinity of **1b** molecule weaker. Therefore, this allows compounds **3a** and **3c** to be synthesized directly in anhydrous ethanol, while the synthesis of compound **3b** requires the addition of piperidine as a catalyst [48].

**Figure 1:**
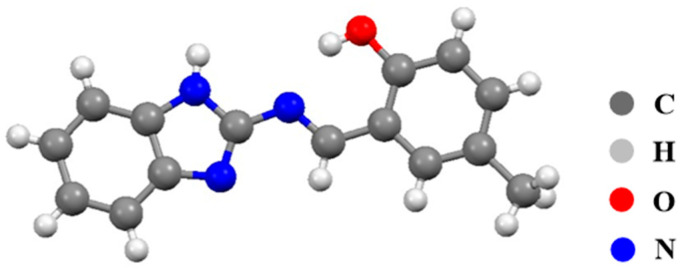
Crystal structure of compound **3a**.

From the characterization spectra of ^1^H NMR and ^13^C NMR of the synthesized compounds, it can be observed that compounds **3a**–**3c** can find the corresponding peaks at the expected chemical shifts. In addition, the HRMS and X-ray single crystal diffraction of compound **3a** (Table S1) further confirmed its structure (Figure 1). In a word, various test characterizations indicate that the synthesized compounds **3a**–**3c** are indeed the target compounds, as designed.

### 2.2. Theoretical Calculation Study for Compounds ***3a**–**3c***

As is well known, the excited-state intramolecular proton transfer (ESIPT) refers to the process of transferring protons from proton donor groups to nearby proton acceptor groups after a molecule transitions from the ground state to the excited state [49]. Salicylaldehyde Schiff base derivatives can use phenolic hydroxyl groups as proton donors and nitrogen atoms in imines as proton acceptors, thereby generating significant ESIPT driving forces [50].

Based on this, we designed and synthesized Schiff base probe molecules **3a**–**3c** by using 5-methyl salicylaldehyde (**2**) and nitrogen-containing heterocycles (**1a**–**1c**) as the starting materials. When the probe coordinates with metal ions, the ESIPT effect is turned off, and the fluorescence of the probe changes, making it suitable for detecting metal ions. Compounds with the ESIPT effect often have ketone and enol isomers [49]. To demonstrate the ESIPT effect of probes **3a**–**3c**, we calculated the highest occupied molecular orbital (HOMO) and the lowest unoccupied molecular orbital (LUMO) energies of the enol and ketone isomers of probes **3a**–**3c**.

**Figure 2:**
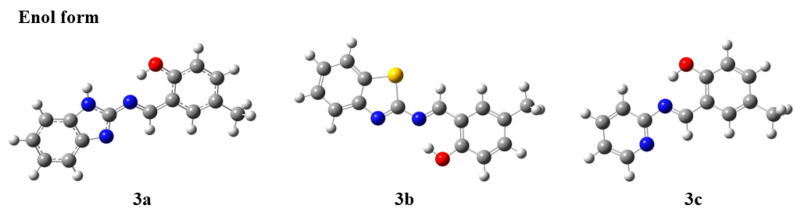
Optimized molecular structures of compounds **3a**–**3c** (enol form).

**Figure 3:**
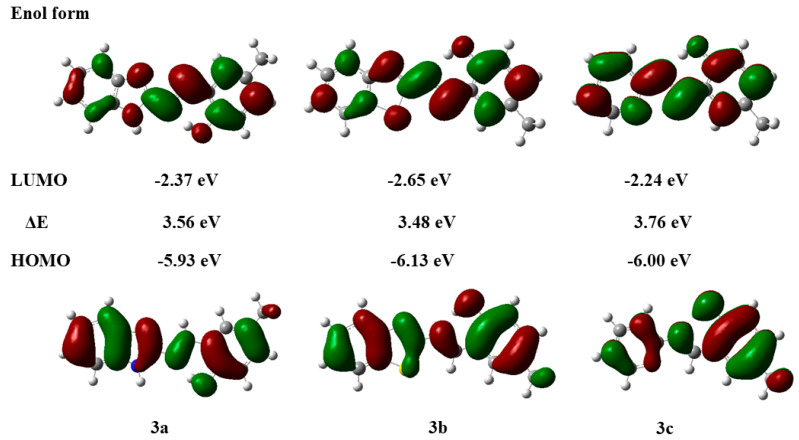
Frontline molecular orbitals and energy levels of compounds **3a**–**3c** (enol form).

**Figure 4:**
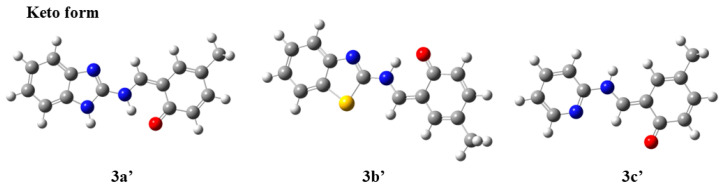
Optimized molecular structures of compounds **3a′**–**3c′** (keto form).

**Figure 5:**
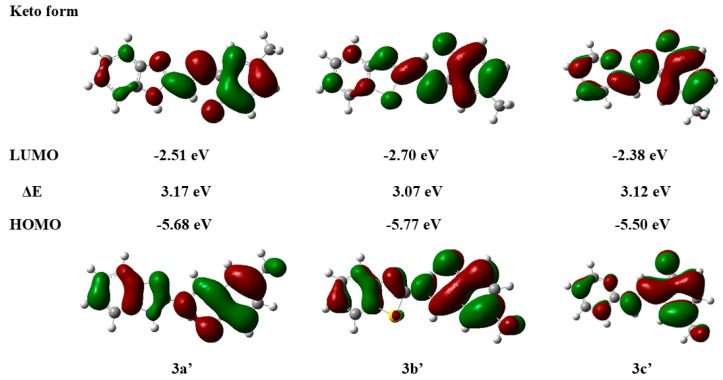
Frontline molecular orbitals and energy levels of compounds **3a′**–**3c′** (keto form).

Based on the Gaussian 09 program D.01, time-dependent DFT (TD-DFT) calculations were performed on these synthesized compounds **3a**–**3c** and their keto tautomers by using the B3LYP/6-31G(d,p) basis set, following the method described in references [51,52]. The aim is to analyze the relationship between structure and photophysical properties, as well as to investigate the ESIPT properties of compounds **3a**–**3c** [53]. The optimized molecular structures and their corresponding frontier molecular orbitals are shown in Figure 2 and Figure 3 (enol form) and Figure 4 and Figure 5 (ketone form), respectively.

After geometry structural optimization, all compounds **3a**–**3c** exhibit near-planar structure, as shown in Figure 2, which is favorable for molecular aggregation. Meanwhile, as shown in Figure 3, there is a large steric overlap between the HOMO and LUMO of compounds **3a**–**3c**, which are widely distributed throughout the probe molecule. This indicates the good fluorescence properties of probes. However, the fast isomerization around the C=N bond weakens the fluorescence properties of compounds **3a**–**3c**, which may be attributed to their ESIPT process [39].

As shown in Figure 4, the optimized geometric structures of the ketone isomers of compounds **3a**–**3c** still exhibit near-planar structure. Furthermore, we calculated the energy states generated by the HOMO and LUMO orbitals of the ketone isomers of compounds **3a**–**3c** (Figure 5). It can be seen that the LUMO energy level of the enol isomers is higher than that of the ketone isomers. Therefore, the enol isomers are unstable in the excited state, and the phenolic hydroxyl proton will transfer to the nitrogen atom of the Schiff base, forming ketone isomers. Meanwhile, the energy gaps of the ketone isomers of compounds **3a**–**3c** (∆E’_3a_ = 3.17 eV, ∆E’_3b_ = 3.07 eV, ∆E’_3c_ = 3.12 eV) are smaller than those of the enol isomers (∆E_3a_ = 3.56 eV, ∆E_3b_ = 3.48 eV, ∆E_3c_ = 3.76 eV), laying the foundation for the formation of ESIPT effect [44].

### 2.3. Study on the Optical Properties of Compounds ***3a**–**3c***

#### 2.3.1. Study on the Optical Properties of Compounds **3a**–**3c** in Different Solvents

To better investigate the performance of probes, we studied the fluorescence emission spectra of compounds **3a**–**3c** in different solvents (Figure S1). It can be seen that the compound has two emission peaks in lower polarity solvents (DCM, EA) for all three probes, belonging to the enol/ketone form, which is consistent with the ESIPT effect [54], and it is obvious that, in DMF, there is the best fluorescence signal. Therefore, in order to obtain a better luminescence intensity while taking into account the solubility problem, we chose to observe the fluorescence detection ability of compounds **3a**–**3c** in DMF.

#### 2.3.2. Study on the ACQ Properties of Compounds **3a**–**3c**

**Figure 6:**
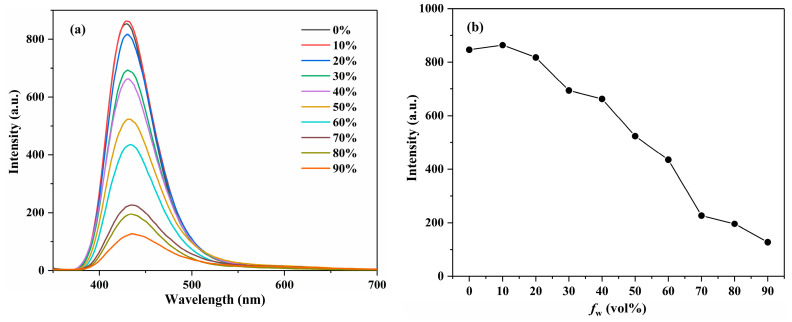
(**a**) The fluorescence spectra of compound **3c** (10 μM) and (**b**) the plot of emission peak intensity in DMF/H_2_O systems with different water fractions (0–90% by volume).

We studied the fluorescence properties of compounds **3a**–**3c** in solvent systems of DMF and water at different ratios using fluorescence emission spectroscopy. The fluorescence spectra are shown in Figures S2 and S3 and Figure 6. The compounds **3a**–**3c** have similar structures, and their fluorescence emission peaks exhibit the same trend in solvent systems of DMF and water at different ratios.

It can be seen that compared with pure DMF solution, the fluorescence emission peak slightly red-shifts when water is added, and the fluorescence intensity is highest when the water ratio is 10%. Then, as the water ratio continues to increase, the fluorescence intensity decreases significantly. This may be due to the aggregation of compounds with increasing water ratio, resulting in an aggregation-caused quenching (ACQ) effect [55] and a decrease in fluorescence intensity.

Based on the above analysis, a DMF/H_2_O solvent system with a water content of 10% was selected for the subsequent performance research experiments.

### 2.4. Research on the Detection of Metal Ions with Compounds ***3a**–**3c***

#### 2.4.1. Selective Study of Compounds **3a**–**3c** with Metal Ions

**Figure 7:**
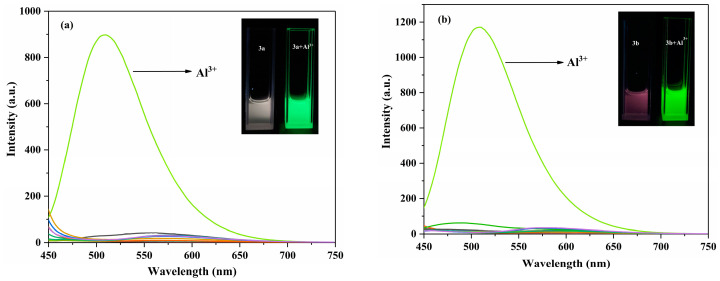
Fluorescence spectra of probe **3a** (**a**) and **3b** (**b**) (10 μM) with various metal ions in DMF/H_2_O (*v*/*v* = 9:1) (λ_ex_ = 405 nm for **3a** and λ_ex_ = 400 nm for **3b**).

The fluorescence emission spectra of compounds **3a**–**3c** (10 μM) and different metal ions (100 μM) were studied in a solvent system of DMF/H_2_O (*v*/*v* = 9:1), including Al^3+^, K^+^, Na^+^, Mg^2+^, Ca^2+^, Zn^2+^, Cu^2+^, Mn^2+^, Co^2+^, Cr^3+^, Hg^2+^, Cd^2+^, Ni^2+^, Pb^2+^, Fe^2+^, and Fe^3+^. The fluorescence spectra are shown in Figure 7.

It can be observed that adding Al^3+^ to the solution of compound **3a** enhances the fluorescence intensity of the emission peak at 508 nm, changing from weak light-yellow fluorescence to yellow-green fluorescence (Figure 7a). Similarly, adding Al^3+^ to the solution of compound **3b** enhances the fluorescence intensity at 509 nm, changing from weak light red fluorescence to strong yellow-green fluorescence (Figure 7b). These may be attributed to the coordination of compounds **3a** and **3b** with Al^3+^, further enhancing their structural rigidity and inhibiting ESIPT and C=N isomerization processes. In addition, the fluorescence response of compounds **3a** and **3b** to other metal ions is almost negligible. Compared with blank probes, the test results indicate that both **3a** and **3b** can serve as a specific “turn-on” probe for Al^3+^.

Differently, both the addition of Al^3+^ and Zn^2+^ to compound **3c** solution can enhance the fluorescence emission peak. Among them, the addition of Al^3+^ causes the solution to change from light purple fluorescence to yellow-green fluorescence while adding Zn^2+^ causes the solution to turn yellow fluorescence, and the fluorescence enhancement is more significant (Figure S4). Of course, the fluorescence response of compound **3c** to other metal ions is almost negligible, also indicating that compound **3c** can serve as a specific “turn-on” probe for Al^3+^ and Zn^2+^.

#### 2.4.2. Anti-Interference Study of Compounds **3a**–**3c** with Metal Ions

In order to further evaluate the selectivity of compounds **3a**–**3c** towards target ions, competitive experiments were conducted in a solvent system of DMF/H_2_O (*v*/*v* = 9:1) to investigate the anti-interference of compounds **3a**–**3c**.

**Figure 8:**
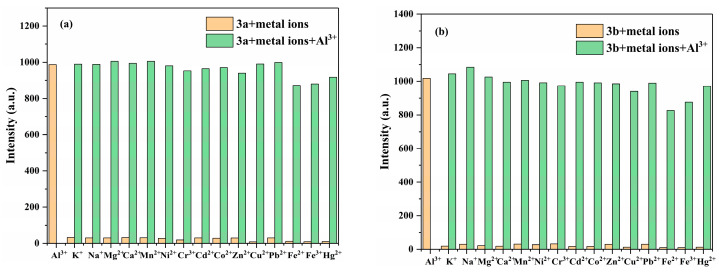
Fluorescence intensity of probe **3a** (**a**) and **3b** (**b**) (10 μM) with Al^3+^ (10 eq.) and solutions after adding Al^3+^ (10 eq.) ions upon the addition of various metal ions (10 eq.) in DMF/H_2_O (*v*/*v* = 9:1) (λ_ex_ = 405 nm for **3a** and λ_ex_ = 400 nm for **3b**).

From Figure 8, it can be seen that the fluorescence is enhanced when Al^3+^ is added to compounds **3a** (Figure 8a) and **3b** (Figure 8b). On the contrary, the fluorescence intensity of the solution does not change significantly when other metal ions are added. However, after continuing to add Al^3+^, the fluorescence is enhanced. These indicate that most metal ions do not significantly interfere with the detection of Al^3+^ for compounds **3a** and **3b**.

Similarly, as shown in Figure S5a, the fluorescence is enhanced when Al^3+^ is added to compound **3c**, while the fluorescence intensity of the solution does not change significantly when other metal ions (excluding Zn^2+^) are added. However, after continuing to add Al^3+^, the fluorescence is enhanced. In addition, after adding Al^3+^ to compound **3c** and continuing to add Zn^2+^, the fluorescence intensity did not change significantly, indicating that the complex formed between compound **3c** and Al^3+^ is more stable. Thus, other metal ions (including Zn^2+^) have no significant interference with the detection of Al^3+^ for compound **3c**.

Notably, as shown in Figure S5b, there is a difference in that the fluorescence is enhanced when Zn^2+^ is added to compound **3c**, while the fluorescence intensity of the solution does not change significantly when other metal ions (excluding Al^3+^) are added. After continuing to add Zn^2+^, some solutions (previously added with K^+^, Na^+^, Mg^2+^, Ca^2+^, Cu^2+^, Mn^2+^, Co^2+^, Cd^2+^, Ni^2+^ solutions) show the enhanced fluorescence. However, after adding Zn^2+^ to the solution previously containing Cr^3+^, Hg^2+^, Pb^2+^, Fe^2+^, and Fe^3+^, there is no significant change in the fluorescence of the solution. These indicate that the above-mentioned metal ions can interfere with the detection of Zn^2+^ for compound **3c**.

#### 2.4.3. Quantitative Identification of Al^3+^ by Compounds **3a**–**3c**

Probes **3a**–**3c** exhibit excellent selectivity towards Al^3+^. Furthermore, the fluorescence titration experiments were conducted on **3a**–**3c** for Al^3+^, and the changes in fluorescence emission curves were analyzed. Once the fitting curves of the maximum emission intensity and ion concentration of the probe are plotted, and the limit of detection (LOD) can be calculated using the formula LOD = 3δ/K [56].

**Figure 9:**
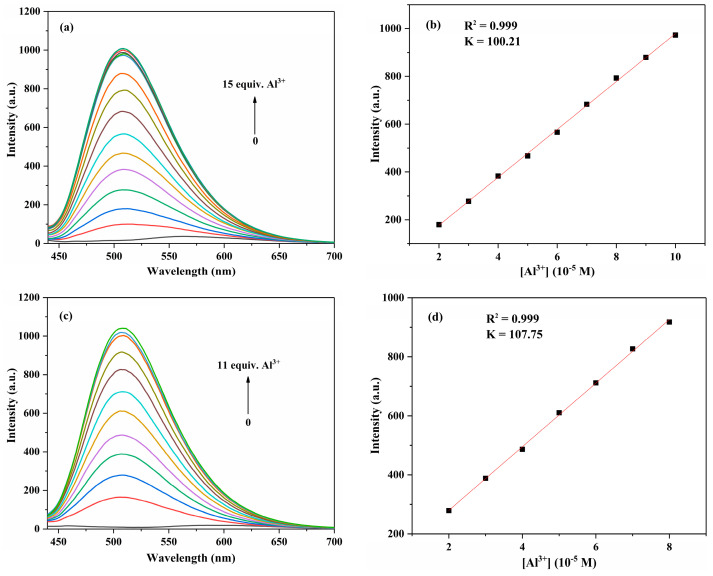
Fluorescence spectra of **3a** (**a**) and **3b** (**c**) in the presence of increasing amounts of Al^3+^; Plot of fluorescence intensities of **3a** (**b**) and **3b** (**d**) respectively.

As shown in Figure 9a, with the increase of Al^3+^ concentration from 0 to 15.0 eq., the fluorescence intensity at 509 nm increases with the increase of concentration. From Figure 9b, it can be observed that there is a good linear relationship between the fluorescence intensity of probe **3a** and the concentration of Al^3+^ (R^2^ = 0.999), and the LOD of probe **3a** for Al^3+^ is calculated to be 3.14 × 10^−7^ M.

Similarly, as shown in Figure 9c, as the concentration of Al^3+^ increases from 0 to 11.0 eq., the fluorescence intensity at 508 nm increases with the increase of the concentration. From Figure 9d, it can be observed that there is a good linear relationship between the fluorescence intensity of probe **3b** and the concentration of Al^3+^ (R^2^ = 0.999), and the LOD of probe **3b** for Al^3+^ is calculated to be 2.81 × 10^−7^ M.

For probe **3c**, as shown in Figure S6a, when 0–12.0 eq. of Al^3+^ is added to the solution of probe **3c**, there is a good linear relationship between the fluorescence intensity of probe **3c** and the concentration of Al^3+^ (R^2^ = 0.996), and the LOD of probe **3c** for Al^3+^ is calculated to be 2.86 × 10^−7^ M (Figure S6b).

Notably, the LOD values of probes **3a**–**3c** are all lower than the maximum allowable concentration in drinking water specified by WHO [7]. In addition, when 0–1.0 eq. of Zn^2+^ is added to probe **3c**, there is a good linear relationship between the fluorescence intensity and concentration of probe **3c** (R^2^ = 0.991) (Figure S7a), and its LOD for Zn^2+^ is calculated to be 8.64 × 10^−9^ M (Figure S7b).

Although probe **3c** can achieve nanomolar detection of Zn^2+^, its detection of Zn^2+^ is susceptible to interference, as mentioned above, so in the future, no further research will be conducted on the detection of Zn^2+^.

#### 2.4.4. Study on the Time Response of Probes **3a**–**3c** to Al^3+^

The response speed of the probe to metal ions is an important factor in determining the real-time performance of the proposed fluorescence sensor [57]. Therefore, by rapidly adding 10.0 eq. of Al^3+^ solution to the solution of probes **3a**–**3c** and testing their fluorescence intensity at different times, the relationship between the maximum fluorescence intensity of the probe and time was studied.

**Figure 10:**
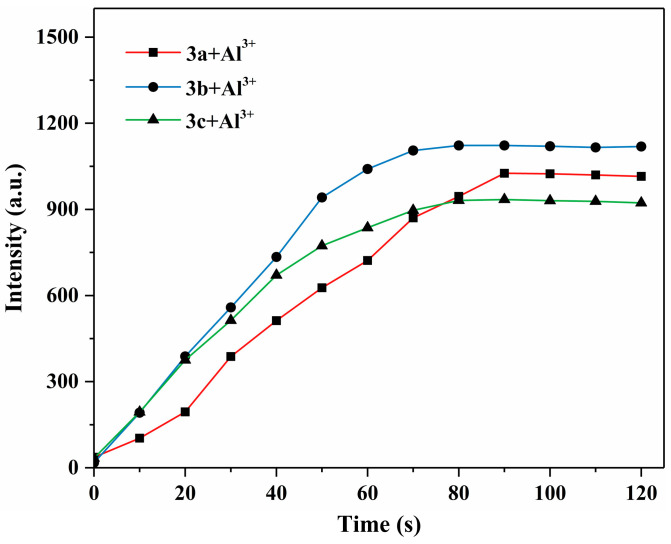
Time-dependent variation of maximum fluorescence intensity after the interaction between compound **3a**–**3c** and Al^3+^.

From Figure 10, it can be observed that after adding Al^3+^ to the compound solution, the fluorescence intensity immediately increases and remains almost unchanged after 100 s. Meanwhile, the reaction times of probes **3b** and **3c** with Al^3+^ are both 80 s, slightly faster than probe **3a** (90 s).

Delightedly, the response times of probes **3a**–**3c** are shorter than most reported Al^3+^ small molecule fluorescent probes [20,21,23] (please see Table S2 for a more detailed comparison). Therefore, the rapid response of compounds **3a**–**3c** to Al^3+^ indicates that these three probes can be used for actual sample detection of Al^3+^.

#### 2.4.5. The Binding Ratio and Binding Constant of Probes **3a**–**3c** Interacting with Al^3+^

In order to determine the binding ratio of Al^3+^ to probes **3a**–**3c**, we conducted the continuous variation experiment to get Job’s plot as the reported method [58]: fixing the total concentration of Al^3+^ and ligands at 10 μM, changing the ratio of metal ions to ligands (0.1–0.9), and plotting the relationship between the maximum fluorescence intensity and the mole fraction of Al^3+^. The value of the inflection point corresponds to the situation where Al^3+^ is perfectly coordinated with the ligand.

**Figure 11:**
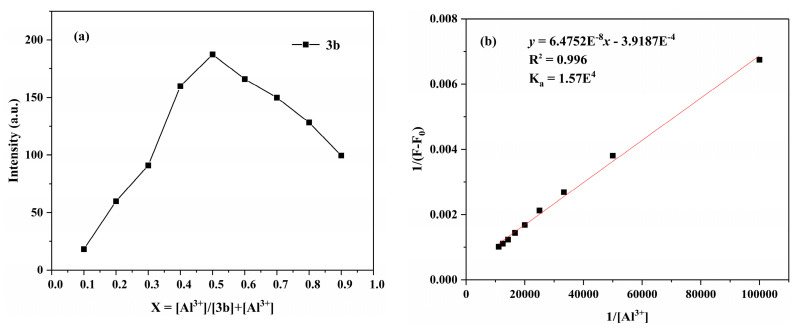
(**a**) The Job plot of **3b**-Al^3+^; (**b**) the Benesi–Hildebrand plot of **3b**-Al^3+^.

As shown in Figure S8a, when the mole fraction of Al^3+^ is 0.5, the Job plot shows a turning point, indicating that the binding stoichiometric ratio of probe **3a** to Al^3+^ forming a complex is 1:1. Furthermore, the fluorescence titration data are linearly fitted by using the Benesi–Hildebrand equation, and the K_a_ value can be calculated by using the intercept and slope of the line [59]. The binding constant between probe **3a** and Al^3+^ is 6.53 × 10^3^ M (Figure S8b). Similarly, as shown in Figure 11a, the maximum fluorescence intensity is achieved when the mole fraction of Al^3+^ is 0.5, indicating that the stoichiometric ratio of probe **3b** to form a complex with Al^3+^ is 1:1. The binding constant between probe **3b** and Al^3+^ is 1.57 × 10^4^ M (Figure 11b), which is higher than most reported Al^3+^ small molecule fluorescent probes [20,29].

For probe **3c**, as shown in Figure S9a, the stoichiometric ratio of compound **3c** to Al^3+^ binding is 1:1. In addition, the binding constant between compound **3c** and Al^3+^ is 7.67 × 10^3^ M (Figure S9b).

#### 2.4.6. Study on pH Tolerance of Probes **3a**–**3c** in Interaction with Al^3+^

**Figure 12:**
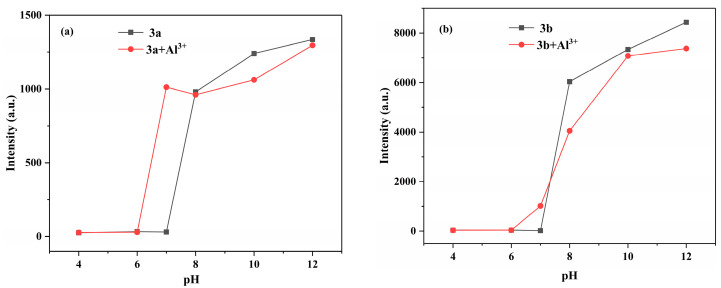
Different pH values of maximum fluorescence intensity before and after the interaction between compound **3a** (**a**)/**3b** (**b**) and Al^3+^.

It is well known that pH has a significant impact on the selectivity and sensitivity of the probes for metal ions [39]. Therefore, we measured and compared the maximum fluorescence intensity before and after the interaction between probes **3a**–**3c** solution and Al^3+^ under different pH environments (Figure 12).

From Figure 12, it can be seen that probes **3a** and **3b** both have weak fluorescence intensity in the acidic range and cannot detect Al^3+^. In other words, probes **3a** and **3b** can only detect Al^3+^ under neutral conditions, also indicating that they can effectively detect the target ion under physiological conditions.

Interestingly, it also can be found that under alkaline conditions, both probes **3a** and **3b** exhibit strong fluorescence. Especially when the pH is 8, the fluorescence intensity of probes **3a** and **3b** will suddenly increase, and it will be enhanced by the increase of alkalinity. This may be due to the hydrolysis of compounds **3a** and **3b** under alkaline conditions [57], and the hydrolysis product 5-methyl salicylaldehyde also exhibits strong fluorescence under alkaline conditions. Especially for probe **3b**, there is also a noticeable color change under the naked eye (Figure S10). Therefore, probes **3a** and **3b** also can be considered as simple alkaline indicators with a mutation point pH of 8.

Differently, as shown in Figure S11, compound **3c** can detect Al^3+^ under neutral and weakly alkaline conditions (no more than pH 8). Perhaps, due to the stronger alkalinity of probe **3c** compared to compounds **3a** and **3b**, it will not be hydrolyzed under the weakly alkaline condition.

However, it also can be found that compound **3c** exhibits strong fluorescence under strong alkaline conditions. When the pH is 9, the fluorescence intensity of compound **3c** suddenly enhances, and it will increase with increasing alkalinity (Figure S11). Thus, considering that probe **3c** also undergoes hydrolysis under strong alkaline conditions and exhibits strong fluorescence (Figure S10), it can also be considered a simple alkaline indicator with a mutation point pH of 9.

In addition, under acidic conditions, the fluorescence of probes **3a**–**3c** did not show significant changes before and after the addition of Al^3+^, and all exhibited no fluorescence. This may be due to the weak fluorescence intensity of the probes caused by the hydrolysis of imine under acidic conditions.

### 2.5. Sensing Mechanism of Compounds ***3a**–**3c*** to Al^3+^

#### 2.5.1. ^1^H NMR and FT-IR Study on the Interaction Between Compounds **3a**–**3c** to Al^3+^

From the previous Job’s plot, it has been known that the stoichiometric ratio for the formation of complexes between compounds **3a**–**3c** and Al^3+^ are all 1:1. In order to further understand the recognition mechanism of probes **3a**–**3c** for Al^3+^, the studies of ^1^H NMR and Fourier transform infrared (FT-IR) spectroscopy (liquid film, using DMF as the solvent) were conducted [29].

**Figure 13:**
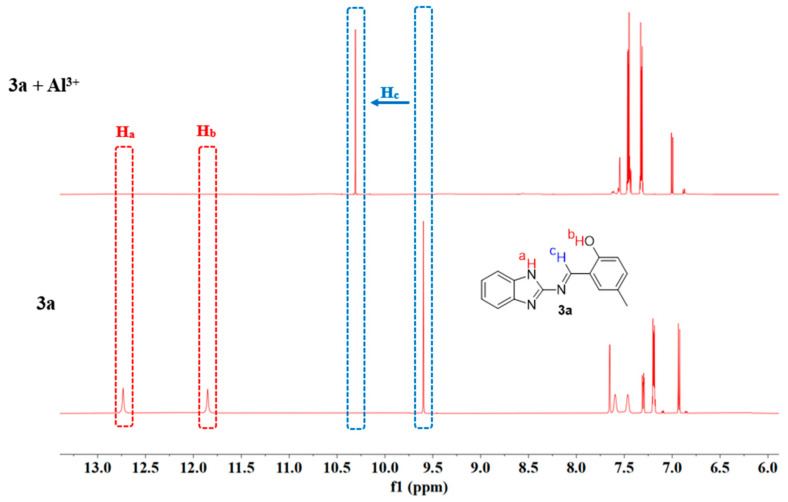
Hydrogen spectra of compound **3a** and **3a** + **Al**^3+^.

The ^1^H NMR spectra of compounds **3a**–**3c** were tested in the absence and presence of Al^3+^ (its nitrate) in DMSO-d_6_. As shown in Figure 13, after the addition of Al^3+^ to compound **3a**, the characteristic imine signal (H_c_) at a chemical shift of 9.60 ppm is shifted to 10.20 ppm, and the amino proton (H_a_) and phenolic hydroxyl proton (H_b_) can not be observed, indicating that the N atom of the amino group, the O atom of the phenolic hydroxyl group, and the N atom of the imine all have participated in the coordination between compound **3a** and Al^3+^ [39].

Similarly, the binding modes of compounds **3b** and **3c** with Al^3+^ can also be obtained through the ^1^H NMR titration experiments (as shown in Figures S12 and S14). Thus, the O atom of the phenolic hydroxyl group and the N atom of the imine in probes **3b** and **3c** can participate in the coordination of compounds **3b** and **3c** with Al^3+^.

**Figure 14:**
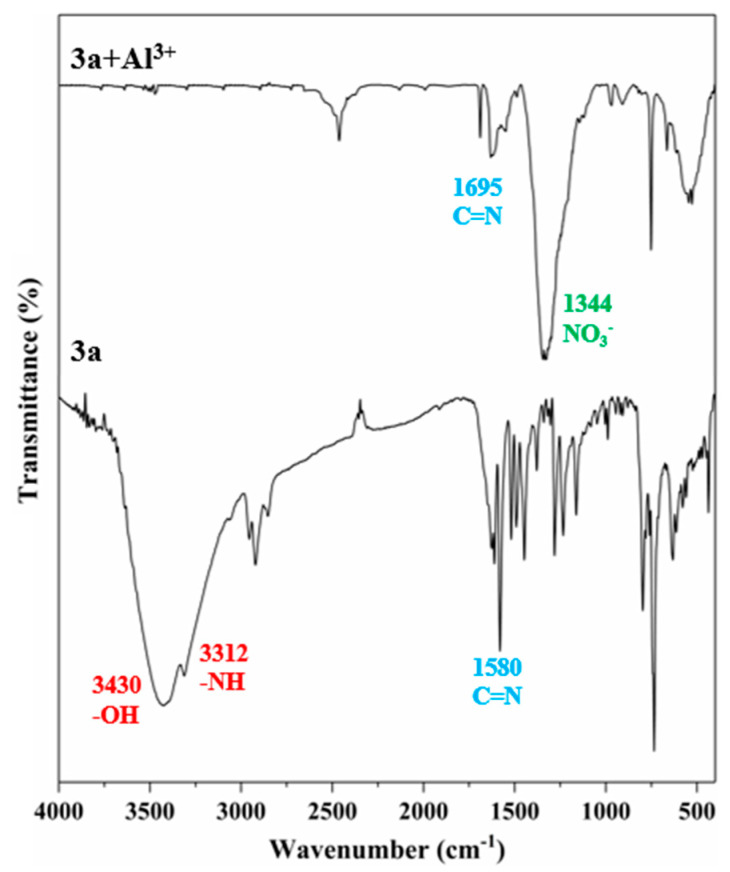
FT-IR spectra of compound **3a** and **3a** + **Al**^3+^.

In the FT-IR spectrum of compound **3a** (Figure 14), there are peaks at 3430 cm^−1^ (-OH) and 3312 cm^−1^ (-NH), while they completely disappeared after the addition of Al^3+^, indicating that the O atom in the phenolic hydroxyl group and the N atom in the amino group are involved in the binding of compound **3a** with Al^3+^. At the same time, the strongest peak of C=N vibration at 1580 cm^−1^ is weakened and shifted to 1695 cm^−1^, indicating that the N atom in the imine group also participates in the binding with Al^3+^ [16]. In addition, a new strong peak appears at 1344 cm^−1^, which represents the stretching frequency of the NO_3_^−^ group [39].

As shown in Figures S13 and S15, the FT-IR spectra of compounds **3b** and **3c** also showed similar results.

#### 2.5.2. DFT Study on the Interaction Between Compounds **3a**–**3c** to Al^3+^

In order to further investigate the coordination interaction between probes **3a**–**3c** and Al^3+^, DFT calculations were conducted on compounds **3a**-Al^3+^, **3b**-Al^3+^, and **3c**-Al^3+^. The possible structures of compounds **3a**–**3c** and Al^3+^ were geometrically optimized, and the LUMO-HOMO energy levels of **3a**-Al^3+^, **3b**-Al^3+^, and **3c**-Al^3+^ were calculated [29].

**Figure 15:**
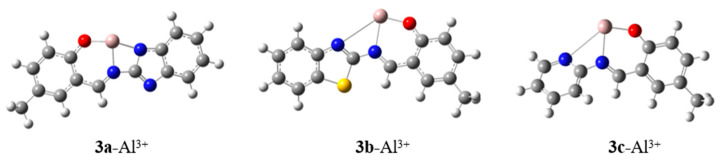
Optimized molecular structures of **3a**-Al^3+^, **3b**-Al^3+^, and **3c**-Al^3+^.

**Figure 16:**
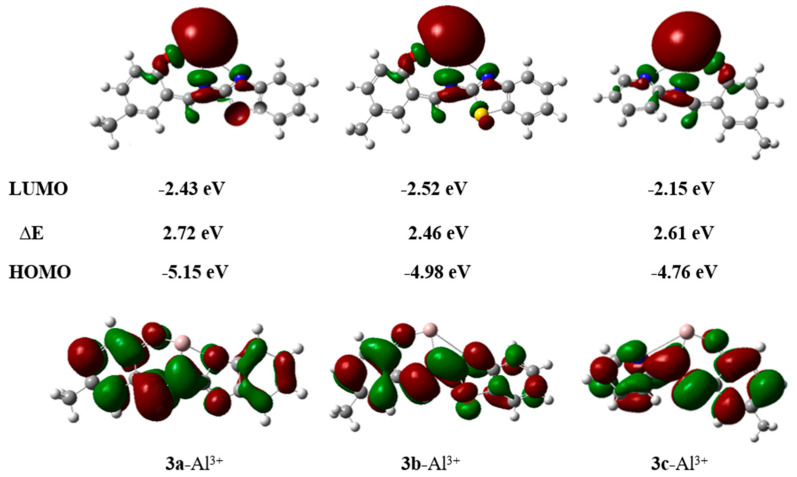
HOMO-LUMO energy level diagram of **3a**-Al^3+^, **3b**-Al^3+^, and **3c**-Al^3+^.

As shown in Figure 15, probes **3a**–**3c** coordinate with Al^3+^ in a stoichiometric ratio of 1:1. When probes **3a**–**3c** coordinate with Al^3+^, the energy of HOMO is mainly distributed in the benzene ring and linker, while the energy of LUMO is mainly distributed near the aluminum atom (Figure 16).

After chelating with aluminum ions, probes **3a**–**3c** exhibit enhanced fluorescence intensity and spectral changes, known as chelation-enhanced fluorescence effect (CHEF) [3]. The CHEF effect significantly increases the fluorescence intensity [17], emitting bright yellow-green fluorescence. The energy gaps of **3a**-Al^3+^, **3b**-Al^3+^, and **3c**-Al^3+^ are 2.72 eV, 2.46 eV, and 2.61 eV, respectively (Figure 16), which are significantly smaller than the energy gaps of compounds **3a**–**3c** (∆E_3a_ = 3.56 eV, ∆E_3b_ = 3.48 eV, ∆E_3c_ = 3.76 eV). These indicate that compounds **3a**–**3c** tend to bind with Al^3+^ and form more stable complexes [21,42,60]. The molecule’s stiffness decreases non-radiative decay processes, further verifying that compounds **3a**–**3c** and Al^3+^ “turn on” fluorescence through the CHEF effect.

In addition, compared with **3a**-Al^3+^ and **3c**-Al^3+^, **3b**-Al^3+^ has the lowest energy gaps (Figure 16). This outcome finely explains why probe 3b can recognize Al^3+^ with more obvious fluorescence changes and may correspond to the faster detection time [61].

**Scheme 3:**
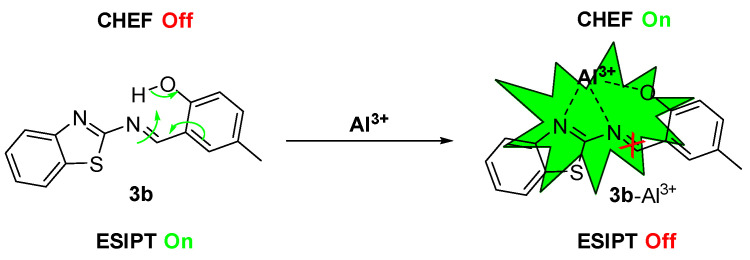
Proposed mechanism of compound **3b** for detection of Al^3+^.

At the same time, combining the ^1^H NMR and FT-IR tests has shown that when coordinating with Al^3+^, the O atom of the phenolic hydroxyl group and the N atom of the imine both participate in the reaction, inhibiting the C=N isomerization and enhancing the rigidity of the probes after complexation with Al^3+^, while the ESIPT effect was turned off and the CHEF effect was activated, resulting in fluorescence enhancement of the probe solution after the addition of Al^3+^ [39]. Therefore, taking probe **3b** as an example with the best testing effect, the detection mechanism of probes **3a**-**3c** can be shown as Scheme 3.

### 2.6. Practical Detection Application of Probes ***3a**–**3c*** to Al^3+^

#### 2.6.1. Application of Probes **3a**–**3c** in Detecting Al^3+^ in Actual Water and Soil Samples

Al^3+^ is prone to accumulate in water and soil samples, posing a serious threat to human health [7]. In order to evaluate the feasibility of probes **3a**–**3c** on Al^3+^ in actual samples, we took distilled water, tap water, river water (the Pearl River water), and three actual water samples as well as soil samples as research objects (the geographical coordinates of all samples: latitude 23.052594° North and longitude 113.380105° East), and used the spiking method to conduct fluorescence emission spectrum test and fluorescence analysis to calculate the content of Al^3+^ in the samples. The recovery rate and relative standard deviation (RSD) were determined through 3 parallel tests [60,62].

As shown in Figure S16, adding different concentrations (10 μM, 20 μM, 40 μM) of Al^3+^ to different samples will gradually increase the fluorescence intensity of probe **3a** with the increase of Al^3+^ concentration. According to Table S3, the recovery range of probe **3a** for low concentration Al^3+^ (10^−5^ M) is 95.5–107%, with RSD less than 3.16%. These results indicate that probe **3a** exhibits high accuracy in identifying Al^3+^ in actual samples and is suitable for detecting Al^3+^ in actual water and soil samples.

**Figure 17:**
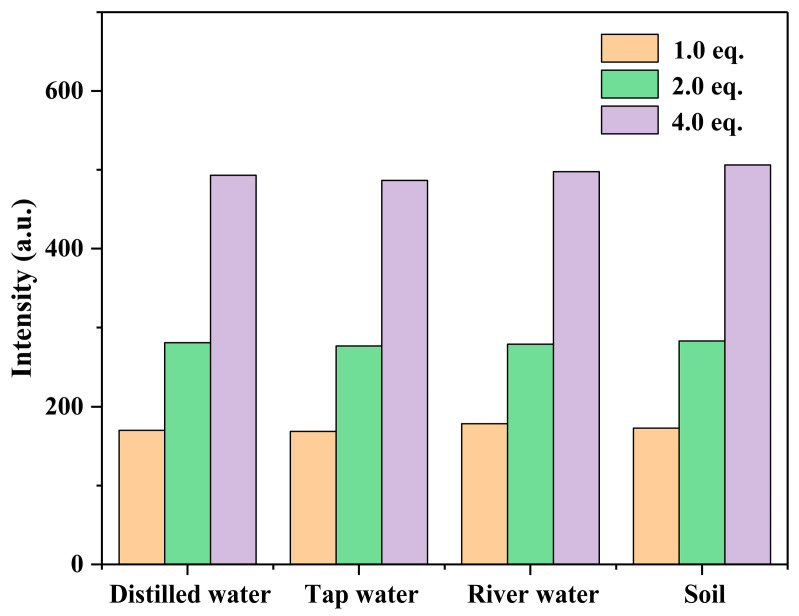
Relationship between the maximum fluorescence intensity of compound **3b** in different water and soil samples with different concentrations of Al^3+^.

Similarly, as shown in Figure 17, the fluorescence intensity of probe **3b** gradually increases with the increase of Al^3+^ concentration. Meanwhile, according to Table 1, the recovery rate range of probe **3b** for low concentration Al^3+^ (10^−5^ M) is 98–105%, with RSD less than 3.09%. Therefore, these results indicate that probe **3b** is also suitable for detecting Al^3+^ in actual water and soil samples.

In addition, the fluorescence intensity of probe **3c** is positively correlated with the concentration of Al^3+^ (Figure S17), and the recovery range of compound **3c** for Al^3+^ (10^−5^ M) is 97–105%, with RSD less than 2.67% (Table S4). Therefore, these results indicate that probe **3c** is also suitable for detecting Al^3+^ in actual water and soil samples.

In a word, compounds **3a**–**3c,** as three probes, are practical for detecting Al^3+^ in actual water and soil samples.

#### 2.6.2. Application of Test Strips Loaded with Compounds **3a**–**3c** for the Detection of Al^3+^

The detection of metal ions through test strips has the advantages of convenience, simple operation, and low cost [44,63]. Therefore, in order to expand the practical applications of probes **3a**–**3c**, strip experiments were conducted [64,65] by cutting the Whatman filter paper into appropriate sizes, soaking them in DMF solution (10^−3^ M) of compounds **3a**–**3c** for 10 min, and drying the test strips in an oven to prepare convenient test strips loaded with probes **3a**–**3c**.

**Figure 18:**
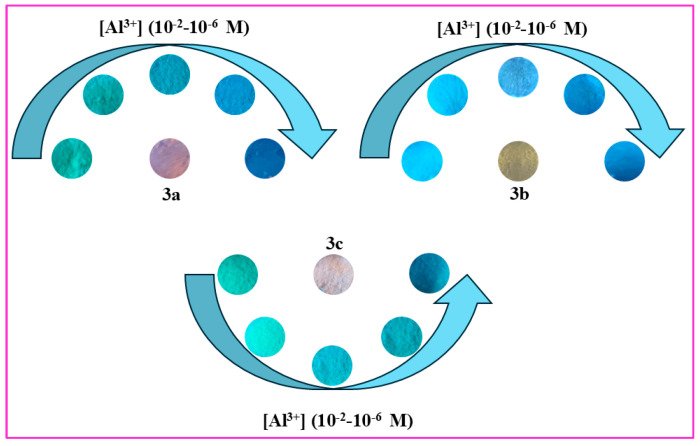
Application of probes **3a**–**3c** in test papers.

Using one group as a blank control, different concentrations of Al^3+^ solution (10^−2^–10^−6^ M) were added dropwise to the other five test strips. After drying with a hair dryer, the test strips were observed under a 365 nm UV lamp, as shown in Figure 18.

Obviously with the increase of Al^3+^ concentration, the fluorescence of the test strips is changed significantly, from blue-green to blue fluorescence. Therefore, probes **3a**-**3c** can be made into test strips for convenient and intuitive detection of Al^3+^.

## 3. Materials and Methods

### 3.1. Reagents and Instruments

2-Aminobenzimidazole (**1a**), 2-aminobenzothiazole (**1b**), 2-aminopyridine (**1c**), and 5-methyl salicylaldehyde (**2**) were all purchased from Anhui Ze Sheng Technology Co., Ltd. (Shanghai, China). Whatman filter paper (110 mm) was purchased from Shanghai Bitai Biotechnology Co., Ltd. (Shanghai, China). All reagents and solvents used in this study are AR grade.

All fluorescence spectra were performed on an F-4600 fluorescence spectrometer (HITACGI Corporation, Tokyo, Japan). FT-IR spectroscopy was obtained using a Fourier transform infrared spectrometer (German Platinum Elmer Spectrum Two model, Ettlingen, Germany). The ^1^H NMR and ^13^C NMR spectra were tested on a 600 MHz Fourier transform nuclear magnetic spectrometer (Bruker AVANCE NEO 600M model, Ettlingen, Germany). Melting points were obtained on the WRX-4 micro melting point instrument (Shanghai Yimenshan Instrument and Equipment Co., Ltd., Shanghai, China). Mass spectrometry was tested on a liquid chromatography high-resolution mass spectrometer (Thermos Fisher Q Exactive, Waltham, MA, USA). X-ray single-crystal diffraction was tested using an X-ray single-crystal diffractometer (Agilent Gemini Model E, Santa Clara, CA, USA).

### 3.2. Synthesis

#### 3.2.1. Synthesis of Compound **3a**

For Scheme 2a, using the method described in reference [66], 2 mmol of 2-amino- benzimidazole (**1a**) and 2 mmol of 5-methyl salicylaldehyde (**2**) were dissolved into 10 mL of anhydrous ethanol in a reaction flask. The reaction was carried out at 80 °C for 3 h. After the reaction was completed, the mixture was cooled to room temperature, giving solid precipitates. After filtration and washing with anhydrous ethanol, a solid crude product was obtained. Then, the further recrystallization with anhydrous ethanol gave the pure product **3a** as a yellow solid with a yield of 91%; m.p.: 235.0–235.5 °C; ^1^H NMR (600 MHz, DMSO-d_6_), δ, ppm: 2.29 (s, 3H), 6.93 (*d*, *J* = 8.4 Hz 1H), 7.16–7.23 (m, 2H), 7.30 (*d*, *J* = 8.4 Hz, 1H), 7.46 (*d*, *J* = 8.4 Hz, 1H), 7.60 (*d*, *J* = 8.4 Hz, 1H), 7.66 (s, 1H), 9.60 (s, 1H), 11.84 (s, 1H), 12.73 (s, 1H), see Figure S18; ^13^C NMR (150 MHz, DMSO-d_6_), δ, ppm: 19.92, 111.22, 116.74, 118.60, 119.09, 121.94, 122.26, 128.28, 131.80, 134.02, 135.54, 142.36, 154.06, 158.45, 165.25, see Figure S19; ESI-MS, *m*/*z* (%): Calcd for C_15_H_14_N_3_O([M + H]^+^): 252.1131 (100), Found: 252.1126 (see Figure S20).

#### 3.2.2. Synthesis of Compound **3b**

For Scheme 2b, using the method described in reference [67], 2 mmol of 2-aminobenzothiazole (**1b**) and 2 mmol of 5-methyl salicylaldehyde (**2**) were dissolved into 5 mL of toluene in a reaction flask. After adding 30 μL of piperidine, the reaction was carried out at 110 °C for 3 h. Once the reaction was completed, the mixture was cooled to room temperature. After adding petroleum ether, there were solid precipitates. After filtration and washing with anhydrous ethanol, a solid crude product was obtained. Then, the further recrystallization with anhydrous ethanol gave the pure product **3b** as a yellow solid with a yield of 85% (51% [68]); m.p.: 131.0–132.4 °C; ^1^H NMR (600 MHz, DMSO-d_6_), δ, ppm: 2.28 (s, 3H), 6.94 (*d*, *J* = 8.4 Hz, 1H), 7.33 (*d*, *J* = 8.4 Hz, 1H), 7.40–7.45 (m, 1H), 7.50–7.55 (m, 1H), 7.73 (s, 1H), 7.94 (*d*, *J* = 8.4 Hz, 1H), 8.07 (*d*, *J* = 8.4 Hz, 1H), 9.39 (s, 1H), 11.24 (s, 1H), see Figure S21; ^13^C NMR (150 MHz, DMSO-d_6_), δ, ppm: 19.90, 116.86, 119.31, 122.39, 122.56, 125.24, 126.70, 128.54, 130.41, 133.94, 136.59, 151.25, 158.57, 165.53, 170.62 (see Figure S22).

#### 3.2.3. Synthesis of Compound **3c**

As Scheme 2c, 2 mmol of 2-aminopyridine (**1c**) and 2 mmol of 5-methyl salicylaldehyde (**2**) were dissolved into 10 mL of anhydrous ethanol in a reaction flask. The reaction was carried out at 80 °C for 1 h. After the reaction was completed, the mixture was cooled to room temperature, giving solid precipitates. After filtration and washing with anhydrous ethanol, a solid crude product was obtained. Then, the further recrystallization with anhydrous ethanol gave the pure product **3c** as an orange solid with a yield of 82% (63% [68]); m.p. 116.5–117.9 °C; ^1^H NMR (600 MHz, DMSO-d_6_), δ, ppm: 2.28 (s, 3H), 6.90 (*d*, *J* = 8.4 Hz, 1H), 7.27 (*d*, *J* = 8.4 Hz, 1H), 7.33–7.39 (m, 1H), 7.44 (*d*, *J* = 7.8 Hz, 1H), 7.57 (s, 1H), 7.89–7.95 (m, 1H), 8.53 (*d*, *J* = 6.0 Hz, 1H), 9.43 (s, 1H), 12.73 (s, 1H), see Figure S23; ^13^C NMR (150 MHz, DMSO-d_6_), δ, ppm: 19.91, 116.58, 118.71, 119.69, 122.84, 127.96, 132.80, 134.91, 139.01, 149.00, 157.69, 158.69, 164.26 (Figure S24).

### 3.3. Computational Details

DFT and TD-DFT calculations were performed using Gaussian 09 D.01, and the structures of probes **3a**–**3c** before and after binding with Al^3+^ were optimized at the B3LYP/6-31G (d) level. Then, their HOMO and LUMO were calculated at the B3LYP/6-31G (d, p) level, and the bandgap value was finally calculated [51,52].

### 3.4. Spectrophotometric Experiments

Firstly, 1 mL of compound **3a**–**3c** solution with a concentration of 1.0 × 10^−2^ M as the mother liquor was prepared and further diluted 1000 times during testing to prepare a test solution of compound **3a**–**3c** with a concentration of 1.0 × 10^−5^ M. Then, serial 10 mL of metal nitrate solutions of Al^3+^, K^+^, Na^+^, Mg^2+^, Ca^2+^, Zn^2+^, Cu^2+^, Mn^2+^, Co^2+^, Cr^3+^, Hg^2+^, Cd^2+^, Ni^2+^, Pb^2+^, Fe^2+^, and Fe^3+^ with the concentration of 1.0 × 10^−2^ M were configured for use.

Fluorescence experiments were conducted to evaluate sensitivity by gradually increasing the concentration of target metal ions in the probe solution. In addition, in fluorescence measurement, the excitation wavelengths of compounds **3a**–**3c** are 405, 400, and 400 nm, respectively. The slit widths of both the excitation and emission were 5.0 nm. All the experiments were performed at room temperature.

## 4. Conclusions

Starting with 5-methylsalicylaldehyde, we designed and synthesized three Schiff base compounds **3a**–**3c** with different nitrogen heterocyclic fluorescent groups through a simple green reaction and characterized them by ^1^H NMR, ^13^C NMR, HRMS, and X-ray single crystal diffraction methods. Based on the coordination interaction between compounds **3a**–**3c** and Al^3+^, compounds **3a**–**3c** have good selectivity for Al^3+^ and can be used as fluorescent probes for detecting Al^3+^. Thus, the properties of three fluorescent probes for Al^3+^ were studied systematically for the first time by combining the theoretical calculations with the experimental observations.

Among them, probe **3b** with benzothiazole as the fluorescent group is the best, with a detection limit of 2.81 × 10^−7^ M and a response time of 80 s. Notably, the LOD values of probes **3a**–**3c** are all below the maximum allowable concentration (7.41 μM) specified by WHO for drinking water. Furthermore, the mechanism of detecting Al^3+^ with probes **3a**-**3c** has been elucidated through ^1^H NMR, FT-IR spectroscopy, and DFT calculation analysis. The theoretical research indicates that the energy gap of compound **3b** is the lowest of the three probes, resulting in its best results. Importantly, these probes **3a**–**3c** can be successfully applied for the practical detection of Al^3+^ in water samples, soil samples, and test strips.

## Figures and Tables

**Table 1:** Determination of Al^3+^ in actual water and soil samples by compound **3b**.

Samples	Al^3+^ Added (10^−5^ M)	Al^3+^ Found (10^−5^ M)	RSD (%, *n* = 3)	Recovery (%)
Distilled water	1	0.98	0.14	98
	2	1.99	1.53	99.5
	4	3.99	1.69	99.8
Tap water	1	0.97	0.88	97
	2	1.97	0.19	98.5
	4	3.92	2.34	98
River water	1	1.05	1.22	105
	2	1.99	3.09	99.5
	4	4.02	2.62	100.5
Soil	1	1.01	0.81	101
	2	2.03	2.30	101.5
	4	4.10	1.35	102.5
Equation	y = 107.75x + 64.17			

## Data Availability

The original contributions presented in this study are included in the article/Supplementary Materials. Further inquiries can be directed to the corresponding author.

## Supplementary Materials

The following supporting information can be downloaded at: www.mdpi.com/article/10.3390/molecules30051128/s1, Data of single-crystal X-ray analysis of compound **3a** (Table S1); Study on the optical properties of compounds **3a**–**3c** in different solvents (Figure S1); Study on the ACQ properties of compounds **3a** and **3b** (Figures S2 and S3); Selective study of compound **3c** with metal ions (Figure S4); Anti-interference study of compound **3c** with metal ions (Figure S5); Quantitative identification of Al^3+^ by probe **3c** (Figures S6 and S7); Comparison of some fluorescent probes for Al^3+^ (Table S2); The binding ratio and binding constant of compounds **3a** and **3c** interacting with Al^3+^ (Figures S8 and S9); Study on pH tolerance of compounds **3a**–**3c** in interaction with Al^3+^ (Figure S10 and S11); ^1^H NMR and FT-IR study on the interaction between compounds **3b** and **3c** to Al^3+^ (Figures S12–S15); Application of compounds **3a** and **3c** in the detection of Al^3+^ in actual samples (Figures S16 and S17, Tables S3 and S4); NMR Spectra and HRMS for compounds **3a**–**3c** (Figures S18–S24); See [2,15,20,21,23,26,27].

## References

[1] Meng S., Liu J.X., Yang Y.Y., Mao S., Li Z. (2024). Lanthanide MOFs based portable fluorescence sensing platform: Quantitative and visual detection of ciprofloxacin and Al^3+^. Sci. Total Environ..

[2] Zou Y.-L., Liu Y.-T. (2024). A novel isophorone-based NIR fluorescent and colormetric probe for Al^3+^ sensing and its application for living cells and plants imaging. Spectrochim. Acta A.

[3] Sun J.Q., Wang Y.G., Wang M.D., Wang H.M. (2024). A bisalicylhydrazone based fluorescent probe for detecting Al^3+^ with high sensitivity and selectivity and imaging in living cells. Spectrochim. Acta A.

[4] Selvan G.T., Babu L., Enoch I.M.V., Srinivasadesikan V., Mariselvam R., Kumar A.R., Li X.S., Tang P.J., Selvakumar P.M., Zhang Z. (2024). Surface-modified gold nanoparticles: A novel chemical probe for precise fluorescent detection of aluminium (Al^3+^) ions; investigating DFT insights and molecular logic gate behaviour. J. Mol. Liq..

[5] Yao B.Y., Zhang J.H., Han M., Liang L., Li X.H., Cai X.H., Leng Y.L. (2023). Two novel “turn on” fluorescent probes for the determination of Al^3+^ and its applications. Inorg. Chim. Acta.

[6] Che H.C., Tian X.K., Wang J.H., Dai C., Nie Y.L., Li Y., Lu L.Q. (2023). A portable and intelligent logic detector for simultaneous and in-situ detection of Al^3+^ and fluoride in groundwater. J. Hazard. Mater..

[7] Zhou L.-S., Zhang X.-Y., Huang Y. (2024). High selective, sensitive, reversible and imaging application sensor for aluminium detection. J. Mol. Struct..

[8] Mortada W.I., Alharthi S. (2021). Preconcentration of aluminum by dual-cloud point extraction and its determination by inductively coupled plasma-optical emission spectrometry. Curr. Anal. Chem..

[9] Mashhadizadeh M.H., Amoli-Diva M. (2013). Atomic absorption spectrometric determination of Al^3+^ and Cr^3+^ after preconcentration and separation on *3*-mercaptopropionic acid modified silica coated-Fe_3_O_4_ nanoparticles. J. Anal. At. Spectrom..

[10] Ham Y.S., Okazaki M., Suzuki S., Nakagawa N. (2003). Determination of total aluminum concentration in soil solution using capillary electrophoresis. Soil Sci. Plant Nutr..

[11] Sharma S., Debnath J., Ghosh K.S. (2023). Method for highly selective, ultrasensitive fluorimetric detection of Cu^2+^ and Al^3+^ by Schiff bases containing o-phenylenediamine and o-aminophenol. Methods.

[12] Yang K., Shi S.B., Han S.L., Wu J.Y., Li S.C., Zhu R., Tai S.D., Zhang K. (2024). Europium-macrocycle based lab-on-a-molecule fluorescent probe and its multianalyte detection of phosphate and aluminum ions. Sens. Actuators B.

[13] Zhang Y., Wang G. (2024). A novel ethylene linkage-based covalent organic framework for turn-on fluorescence sensing for Al^3+^ with excellent selectivity and sensitivity. Int. J. Biol. Macromol..

[14] Ma L., Xia B.Y., Zhang Y.Y., Lv J.X., Lv Y.G., Zhang X.Y. (2024). A highly sensitive fluorescent probe for the detection of Al^3+^ and study of its practical application. J. Mol. Liq..

[15] Hu Y., Lu L.M., Wu Y.X., Li Y.J., Wang F., Chen X.Q., Fu H.Y., She Y.B. (2024). Bifunctional ratiometric fluorescent membrane containing pyrene-based probe for visual monitoring and efficient removal of Al^3+^. Sens. Actuators B.

[16] Liu Y.C., Li N., Zhang Y.L., Wang Y. (2023). Diphenyl imidazole-based fluorescent chemosensor for Al^3+^ and its Al^3+^ complex toward water detection in food products. Food Chem..

[17] Li M., Li N., Shao F., Wang R., Chen M., Liu Y.-J., Zhao Y., Li R. (2024). Synthesis of a super-low detection limit fluorescent probe for Al^3+^ and its application in fluorescence imaging of zebrafish and cells. Spectrochim. Acta A.

[18] Bhiri F., Chemli M., Sakly N., Bahrouni Y., Wazzan N., Majdoub M. (2025). New chromogenic and fluorogenic symmetric carbazole Schiff base-derivative: Synthesis, sensing performances and smartphone-assisted fluorometric assay for selective Al^3+^ detection in drinking water. J. Mol. Struct..

[19] Lei X.L., Zou Y.Y., Liang Q.J., Xu W.S., Lao S.S., Yang B., Li L.G., Yang L.T., Liu H., Ma L.J. (2022). An ultrasensitive *4*-(diethylamino) salicylaldehyd-based fluorescence enhancement probe for the detection of Al^3+^ in aqueous solutions and its application in cells. J. Photochem. Photobiol. A.

[20] Yasar O.G., Elmas S.N.K., Aydin D., Arslan F.N. (2024). Al^3+^ selective ratiometric fluorescent and colorimetric chemoprobe and its practical applications in foods, test kits and smartphone. J. Photochem. Photobiol. A.

[21] Wang S., Liao Y.H., Feng H.J., Wu L.Y., He W.Y. (2024). The studies on a fluorescent probe of Schiff base modified by 1,2,3-triazole to detecting Al^3+^ and living cell imaging. J. Mol. Struct..

[22] Wang X.M., Li Y.F., Liu L.Q., Tan Z.Q., Zhou X.H., You Y.J., Wang S. (2024). A dual-emission fluorescence coordination polymer for simultaneous quantification of Al^3+^ and Pb^2+^ in their mixtures. J. Mater. Chem. C.

[23] Zhao C., Xu H.M., Meng Y.T., Wang Y., Shuang S.M., Dong C. (2023). Anthraquinone-based Schiff base fluorescent probe for the sequential sensing of Al^3+^ and pyrophosphate in a near-perfect aqueous solution and bioimaging. J. Mol. Liq..

[24] Suganthi G., Ajitha R., Babu A.A., Kamalesu S., Subramanian R., Arun T., Godlyn A., Nagaraj K. (2024). Highly efficient fluorescence sensing of Al^3+^ ions using a sensitive carbazole based Schiff base. Inorg. Chim. Acta.

[25] Zhang Z.Y., Wang S., Wang M.X., Li H.M., Liang Q.J., Tang J.W., Sun J., Ma L.J., Liu H. (2024). An ultra-sensitive fluorescence multi-channel and colorimetric probe based on salicylaldehyde hydrazone for Al^3+^ recognition with a 3:1 binding ratio. J. Mater. Chem. C.

[26] Dev K., Singh S., Bhardwaj S., Kukreti P., Ramakanth D., Kumar P., Saini S., Roy P., Srivastava V.C., Ghosh K. (2024). Solvent-selective fluorescence sensing of Mg^2+^ and Al^3+^ ions by pincer-type NNO Schiff base ligand: An experimental and DFT optimized approach. Chem.-Eur. J..

[27] Islam M.S., Hoque A., Baig K.M.Y., Sarmin M., Kole G.K., Hoda M., Alam M.A. (2024). A zwitterionic probe for ratiometric fluorescent detection of aluminium(III) ion in aqueous medium and its application in bioimaging. Spectrochim. Acta A.

[28] Goswami N., Naithani S., Goswami T., Kumar P., Kumar S. (2024). A quinoline derived Schiff base as highly selective ‘turn-on’ probe for fluorogenic recognition of Al^3+^ ion. Spectrochim. Acta A.

[29] Elmas S.N.K., Boran T., Arslan F.N., Özhan G., Erdemir S. (2024). Ultrafast and nanomolar detection of Al^3+^: Furazan based fluorescent chemosensor and its practices in smartphone, test kit, water samples and living-cell. Microchem. J..

[30] Jiang Q., Song J., Yang X.Q., Rao X.P., Zhao P., Wang Z.L. (2024). A novel reversible fluorescent probe for sequential detection of Al^3+^ and HPO_4_^2−^ based on caffeic acid and its applicability in cell imaging. Spectrochim. Acta A.

[31] Chen S.-H., Jiang K., Xiao Y., Cao X.-Y., Arulkumar M., Wang Z.-Y. (2020). Recent endeavors on design, synthesis, fluorescence mechanisms and applications of benzazole-based molecular probes toward miscellaneous species. Dyes Pigm..

[32] Chen S.-H., Jiang K., Liang Y.-H., He J.-P., Xu B.-J., Chen Z.-H., Wang Z.-Y. (2023). Fine-tuning benzazole-based probe for the ultrasensitive detection of Hg^2+^ in water samples and seaweed samples. Food Chem..

[33] Wang R.Y., Feng X., Feng B.Y., Chen Y. (2024). Boron-mediated one-pot access to salicylaldehydes via *ortho*-C-H hydroxylation of benzaldehydes. RSC Adv..

[34] Mohammed A.K., Pandikassala A., Sánchez P.P., Gaber S.A., Canossa S., Kurian M., Xavier G., He Y., Gándara F., Kurungot S. (2024). Iron salicylaldehydate conjugated metal-organic framework for quasi solid-state supercapacitor. Chem. Eng. J..

[35] Mandi A., Bar N., Biswas D., Ray A., Ghosh K., Mondal D., Das G.K., Chowdhury P. (2024). Rhodamine 6G salicylaldehyde hydrazone-Zn complex: Synthesis, characterization, photochromism, understanding, and application. J. Lumin..

[36] Sahu M., Ganguly M., Sharma P. (2024). Recent applications of coinage metal nanoparticles passivated with salicylaldehyde and salicylaldehyde-based Schiff bases. Nanoscale Adv..

[37] Rajalakshmi K., Muthusamy S., Lee H.J., Kannan P., Zhu D.W., Song J.W., Nam Y.S., Heo D.N., Kwon I., Luo Z.B. (2024). Dual-channel fluorescent probe for discriminative detection of H_2_S and N_2_H_4_: Exploring sensing mechanism and real-time applications. J. Hazard. Mater..

[38] Zhou N., Cai M.Y., Zheng S.N., Guo H.Y., Yang F.F. (2024). First organic fluorescent sensor for pesticide paclobutrazol based on tetraphenylimidazole Schiff base. Sens. Actuators B.

[39] Dathees T.J., Narmatha G., Prabakaran G., Seenithurai S., Chai J.D., Kumar R.S., Prabhu J., Nandhakumar R. (2024). Salicylaldehyde built fluorescent probe for dual sensing of Al^3+^, Zn^2+^ ions: Applications in latent fingerprint, *bio*-imaging & real sample analysis. Food Chem..

[40] Palta A., Kumar G., Paul K., Luxami V. (2024). Highly selective colorimetric and fluorescent probe for F^−^ and P_2_O_7_^4−^ based on AIEE and dual ESIPT. J. Mol. Struct..

[41] Dwivedi S.K., Arachchige D.L., Vohs T., Tang J.N., Usimaki K., Olowolagba A.M., Fritz D.R., Luck R.L., Werner T., Liu H.Y. (2023). Near-infrared rhodol dyes bearing salicylaldehyde moieties for ratiometric pH sensing in live cells during mitophagy and under hypoxia conditions. J. Mater. Chem. B.

[42] Yang Y.-Y., Ma P.-Y., Xue J.-H., Yang D.-D., Shi Y.-S., Zhao X., Ma Q. (2024). A highly selective fluorescent probe based on multi-binding site hydrazone chemosensor for Al^3+^ detection. Microchem. J..

[43] Pang Y.L., Meng D.S., Liu J., Duan S.X., Fan J.R., Gao L.Y., Long X.S. (2023). Schiff base compounds as fluorescent probes for the highly sensitive and selective detection of Al^3+^ ions. Molecules.

[44] Zhao J.N., Li C.Q., Wei S.H., Lu C.W., Zou L.W. (2023). A multifunctional fluorescent probe based on Schiff base with AIE and ESIPT characteristics for effective detections of Pb^2+^, Ag^+^ and Fe^3+^. Spectrochim. Acta A.

[45] Duan N., Ding L.Y., Yang S.X., Tian H.Y., Sun B.G. (2024). A benzimidazole-based ‘turn-on’ fluorescent probe for highly sensitive detection of Fe^3+^/^2+^: Synthesis, performance, DFT calculations and applications. J. Mater. Chem. C.

[46] Liu Y.L., Li L., Yue M.L., Yang L., Sun F., Xu G.Q., Fu Y., Ye F. (2022). A switch-on fluorescent probe for detection of mesotrione based on the straightforward cleavage of carbon-nitrogen double bond of Schiff base. Chem. Eng. J..

[47] Wang X.Y., Shu J., Ni T., Xu C.X., Tang J.Y., Xu B., Liu X.Q., Zhang K.M., Jiang W.D. (2023). Multiple stimuli-responsive properties of coumarin-salicylaldehyde Schiff bases. Dyes Pigm..

[48] Soliman A.I.A., Sayed M., Elshanawany M.M., Younis O., Ahmed M., El-Dean A.M.K., Abdel-Wahab A.M.A., Wachtveitl J., Braun M., Fatehi P. (2022). Base-free synthesis and photophysical properties of new Schiff bases containing indole moiety. ACS Omega.

[49] Lu C.X., Xu J.W., Song Z., Dai Z.Y. (2023). Advancements in ESIPT probe research over the past three years based on different fluorophores. Dyes Pigm..

[50] Yang M., Mu H.Y., Gao J.A., Zhen Q., Wang X.N., Guan X.T., Li H., Li B. (2023). Screening the optimal probe by expounding the ESIPT mechanism and photophysical properties in vis-HBX with multimodal substitutions. Molecules.

[51] Zhao C., Aziz A., Lu W.J., Xu H.M., Asif M., Shuang S.M., Dong C. (2024). A turn-on anthraquinone-derived colorimetric and fluorometric dual-mode probe for highly selective Hg^2+^ determination and bioimaging in living organisms. J. Hazard. Mater..

[52] Anan J., Fosu E.A., Obuah C., Ainooson M.K., Aniagyei A., Hamenu L., Oppong A., Muller A. (2024). A DFT and TD-DFT studies of the photosensitizing capabilities of thiophene-based dyes. Comput. Theor. Chem..

[53] Zhao J., Li Q., Guo M.L., Yan L., Hu G.X., Zhu L.X., Yin H., Shi Y. (2024). Solvent effects on the ESIPT emission of salicylaldehyde Schiff base derivative: A theoretical reconsideration. J. Mol. Liq..

[54] Queiroz M.H., Alves T.V., Rivelino R., Canuto S. (2024). Influence of solvents and halogenation on ESIPT of benzimidazole derivatives for designing turn-on fluorescence probes. ACS Omega.

[55] Zhang Z.C., Liu C., Lu Y., Zhao W.L., Zhu Q.G., He H.S., Chen Z.J., Wu W. (2024). In vivo fluorescence imaging of nanocarriers in near-infrared window II based on aggregation-caused quenching. J. Nanobiotechnol..

[56] Jiang K., Chen S.-H., Luo S.-H., Pang C.-M., Wu X.-Y., Wang Z.-Y. (2019). Concise design and synthesis of water-soluble fluorescence sensor for sequential detection of Zn(II) and picric acid via cascade mechanism. Dyes Pigm..

[57] Sidqi M.E., Aziz A.A.A., Abolehasan A.E., Sayed M.A. (2022). Photochemical processing potential of a novel Schiff base as a fluorescent probe for selective monitoring of Al^3+^ ions and bioimaging in human cervical cancer HeLa cells. J. Photochem. Photobiol. A.

[58] Zhao C., Xu H.M., Zhang X.R., Meng Y.T., Shuang S.M., Dong C. (2024). Anthraquinone-metal complex fluorescence sensing platform for monitoring PPi mediated by Al^3+^ and bioimaging. J. Mol. Struct..

[59] Sultana T., Mahato M., Tohora N., Ahamed S., Das S.K. (2023). An azine-based chromogenic, fluorogenic probe for specific cascade detection of Al^3+^ and PO_4_^3−^ ions. J. Photochem. Photobiol. A.

[60] Xing Z.Y., Wang J.L., Huang J.H., Chen X.F., Zong Z.A., Fan C.B., Huang G.M. (2022). A significant fluorescence turn-on probe for the recognition of Al^3+^ and its application. Molecules.

[61] Li L., Zhao J.N., Lue C.W. (2024). ESIPT performance bis-Schiff base for multifunctional detection of Pb^2+^, Mg^2+^ and Al^3+^ ions through “turn-on” response and its biological application. Res. Chem. Intermed..

[62] Chen Z.-H., Chen Z.-J., Zeng Y., Liang Y.-T., Guo J.-L., Yang S.-H., Wang Z.-Y. (2025). Multifunctional *N*-fused fluorescent probes for detection of iron ions and nitro explosives. Spectrochim. Acta A.

[63] Mishra S., Mamidi P., Chattopadhyay S., Singh A.K. (2023). Economically viable multi-responsive probes for fluorimetric detection of trace levels of Ga^3+^, Al^3+^ and PPi in near aqueous medium. J. Photochem. Photobiol. A.

[64] Zhao L.-X., He X.-L., Xie K.-B., Hu J.-J., Deng M.-Y., Zou Y.-L., Gao S., Fu Y., Ye F. (2023). A novel isophorone-based fluorescent probe for recognition of Al^3+^ and its bioimaging in cells and plants. Spectrochim. Acta A.

[65] Chen Z.-H., Chen Z.-J., Li W.-X., Zeng Y., Lin J.-Q., Tao G.-S., Wang Z.-Y. (2024). Synthesis of a novel *N*-fused ring based organic molecule probe and its detection of iron. Inorg. Chim. Acta.

[66] Pan W.Y., Yang X.Y., Wang Y.S., Wu L., Liang N., Zhao L.S. (2021). AIE-ESIPT based colorimetric and “OFF-ON-OFF” fluorescence Schiff base sensor for visual and fluorescent determination of Cu^2+^ in an aqueous media. J. Photochem. Photobiol. A.

[67] Sayed M., Kamal A.M., Ahmed M.M., Hassanien R. (2017). Synthesis of some new heterocyclic compounds containing indole moiety. Eur. Chem. Bull..

[68] Jia W.G., Zhang H., Zhang T., Xie D., Ling S., Sheng E.H. (2016). Half-sandwich ruthenium complexes with Schiff-base ligands: Syntheses, characterization, and catalytic activities for the reduction of nitroarenes. Organometallics.

