# Supplementary material for: Schiff Base Compounds Derived from 5-Methyl Salicylaldehyde as Turn-On Fluorescent Probes for Al3+ Detection: Experimental and DFT Calculations
Source: Molecules. 2025 Feb 28;30(5):1128. doi: 10.3390/molecules30051128 (PMC11901710; doi:10.3390/molecules30051128)
Supplement: Supplementary file 1 [file molecules-30-01128-s001.zip › molecules-3491039-supplementary.pdf]

Supplementary Materials

Schiff Base Compounds Derived from 5-Methyl Salicylaldehyde as Turn-On Fluorescent Probes for Al^{3+} Detection: Experimental and DFT Calculations

Huan-Qing Li, Shi-Hang Yang, Yun Li, Wan-Xin Ye, Zi-Yu Liao, Jia-Qian Lu and Zhao-Yang Wang*

School of Chemistry, South China Normal University, Guangzhou Key Laboratory of Analytical Chemistry for Biomedicine, GDMPA Key Laboratory for Process Control and Quality Evaluation of Chiral Pharmaceuticals, Key Laboratory of Theoretical Chemistry of Environment, Ministry of Education, Guangzhou 510006, China; 2022022611@m.scnu.edu.cn (H.-Q.L.); 2024022743@m.scnu.edu.cn (S.-H.Y.); 2024022879@m.scnu.edu.cn (Y.L.); 2024022841@m.scnu.edu.cn (W.-X.Y.); 20212421083@m.scnu.edu.cn (Z.-Y.L.); 20222421008@m.scnu.edu.cn (J.-Q.L.); wangzy@scnu.edu.cn (Z.-Y.W.)

* Correspondence: wangzy@scnu.edu.cn; Tel.: +86-020-3931-0258; Fax: +86-020-3931-0187

Contents

1. Data of single-crystal X-ray analysis of compound **3a** (**Table S1**)..........................[3]
2. Study on the optical properties of compounds **3a-3c** in different solvents (**Figure S1**)..[4]
3. Study on the ACQ properties of compounds **3a** and **3b** (**Figures S2** and **S3**)...............[5-6]
4. Selective study of compound **3c** with metal ions (**Figure S4**)..[7]
5. Anti-interference study of compound **3c** with metal ions (**Figure S5**)..........................[8]
6. Quantitative identification of Al^{3+} by probe **3c** (**Figures S6** and **S7**)......................[9-10]
7. Comparison of some fluorescent probes for Al^{3+} (**Table S2**)......................................[11]
8. The binding ratio and binding constant of compounds **3a** and **3c** interacting with Al^{3+} (**Figures S8** and **S9**)..[12-13]
9. Study on pH tolerance of compounds **3a-3c** in interaction with Al^{3+} (**Figure S10 and S11**)..[14-15]
10. ^{1}H NMR and FT-IR study on the interaction between compounds **3b** and **3c** to Al^{3+} (**Figures S12-S15**)..[16-19]

11. Application of compounds **3a** and **3c** in the detection of Al^{3+} in actual samples (**Figures S16** and **S17**, **Tables S3** and **S4**)..[20-23]

12. NMR Spectra and HRMS for compounds **3a**-**3c** (**Figures S18-S24**)........................[24-27]

References...[28]

1. Data of single-crystal X-ray analysis of compound 3a

Table S1. Crystal data of compound **3a**.

Identification code	**3a**
Empirical formula	$C_{15}H_{13}N_3O$
Formula weight	251.28
Temperature/K	200
Crystal system	monoclinic
Space group	$P2_1$/c
a/Å	5.9009(8)
b/Å	14.914(2)
c/Å	14.369(2)
α/°	90.00
β/°	100.220(14)
γ/°	90.00
Volume/$Å^3$	1244.5(3)
Z	4
$\rho_{calc}g/cm^3$	1.341
μ/mm^{-1}	0.087
F(000)	528.0
Radiation	MoK*α* (*λ* = 0.71073)
2*Θ* range for data collection/ °	5.462 to 58.402
Index ranges	-4 ⩽ h ⩽ 7, -20 ⩽ k ⩽ 15, -19 ⩽ l ⩽ 19
Reflections collected	5625
Independent reflections	2833 [R_{int} = 0.0175, R_{sigma} = 0.0345]
Data/restraints/parameters	2833/0/174
Goodness-of-fit on F^2	1.044
Final R indexes [I>=2*σ* (I)]	R_1 = 0.0487, wR_2 = 0.1151
Final R indexes [all data]	R_1 = 0.0646, wR_2 = 0.1245
Largest diff. peak/hole / e $Å^{-3}$	0.25/-0.17

2. Study on the optical properties of compounds 3a-3c in different solvents

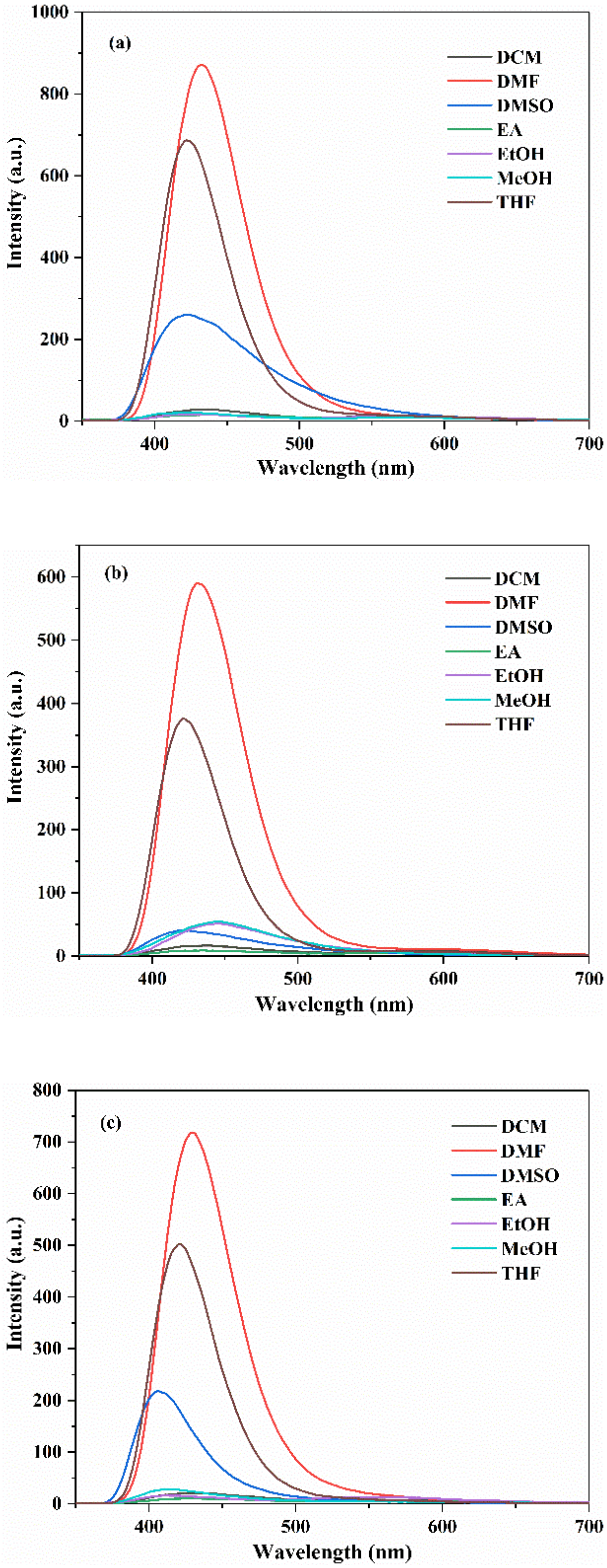

Figure S1. Fluorescence spectra of probes dispersed in different solvents: (a) **3a** (10 μM, λ_{ex}=310 nm); (b) **3b** (10 μM, λ_{ex} = 315 nm); (c) **3c** (10 μM, λ_{ex} = 315 nm).

3. Study on the ACQ properties of compounds 3a and 3b

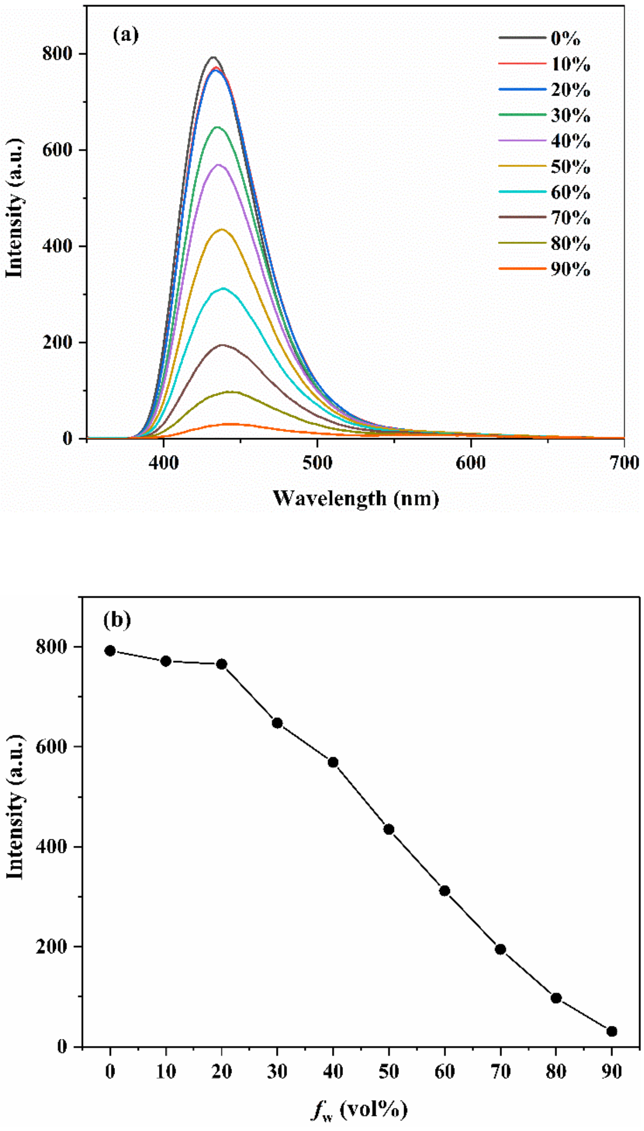

Figure S2. (a) The fluorescence spectra of compound **3a** (10 μM) and (b) plot of emission peak intensity in DMF/H_2O systems with different water fraction (0-90% by volume).

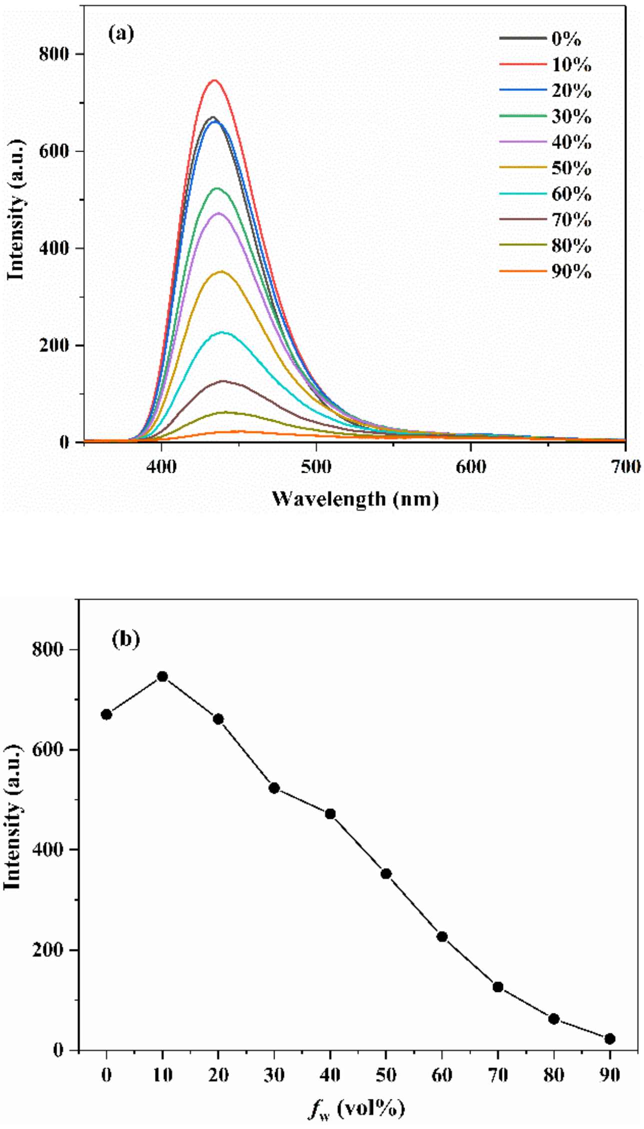

Figure S3. The fluorescence spectra of compound (a) **3b** (10 μM) and plot of emission peak intensity in DMF/H_2O systems with different water fraction (0-90% by volume).

4. Selective study of compound 3c with metal ions

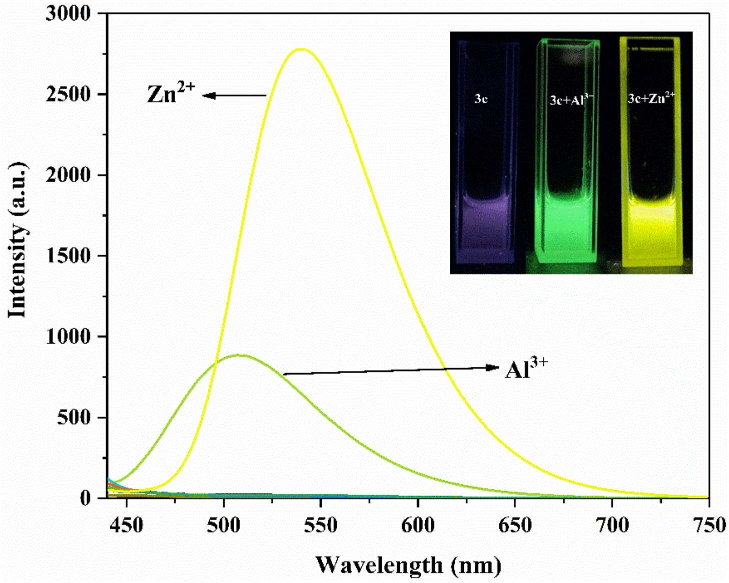

Figure S4. Fluorescence emission spectra of compound **3c** (10 μM) before and after interaction with different metal ions (λ_{ex} = 400 nm); The inset shows the fluorescence changes of compound **3c** before and after adding Al^{3+} or Zn^{2+} (λ_{ex} = 365 nm).

5. Anti-interference study of compound 3c with metal ions

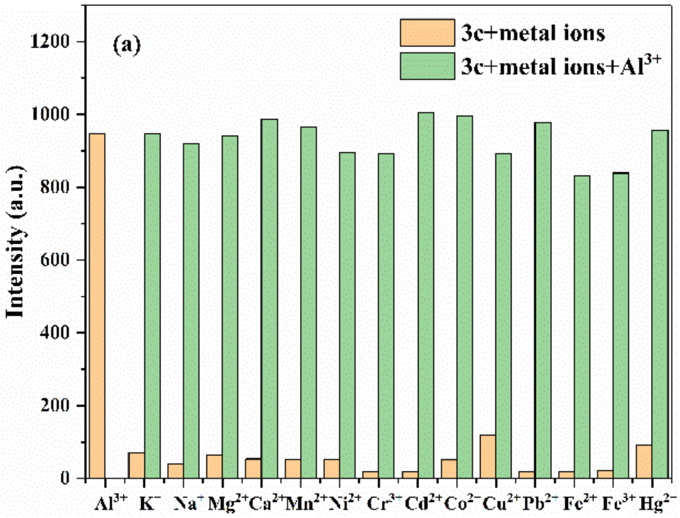

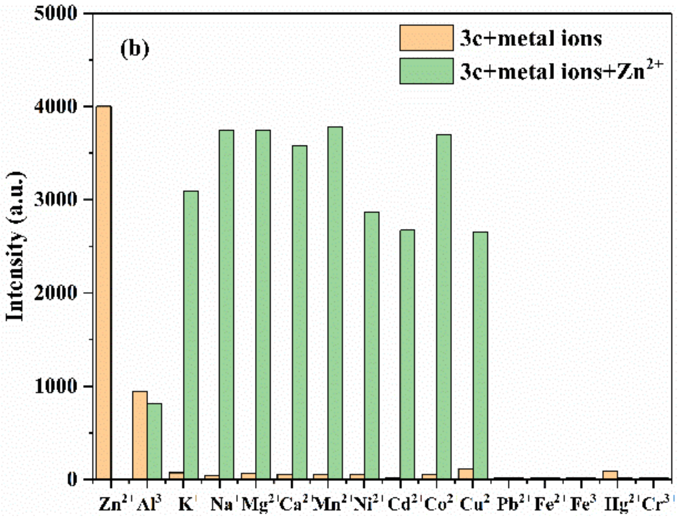

Figure S5. The anti-interference test of compound **3c** for detecting (a) Al^{3+} (Al^{3+}: 10.0 eq.; other metal ions: 10.0 eq.) and (b) Zn^{2+} (Zn^{2+}: 10.0 eq.; other metal ions: 10.0 eq.).

6. Quantitative identification of Al^{3+} by probe 3c

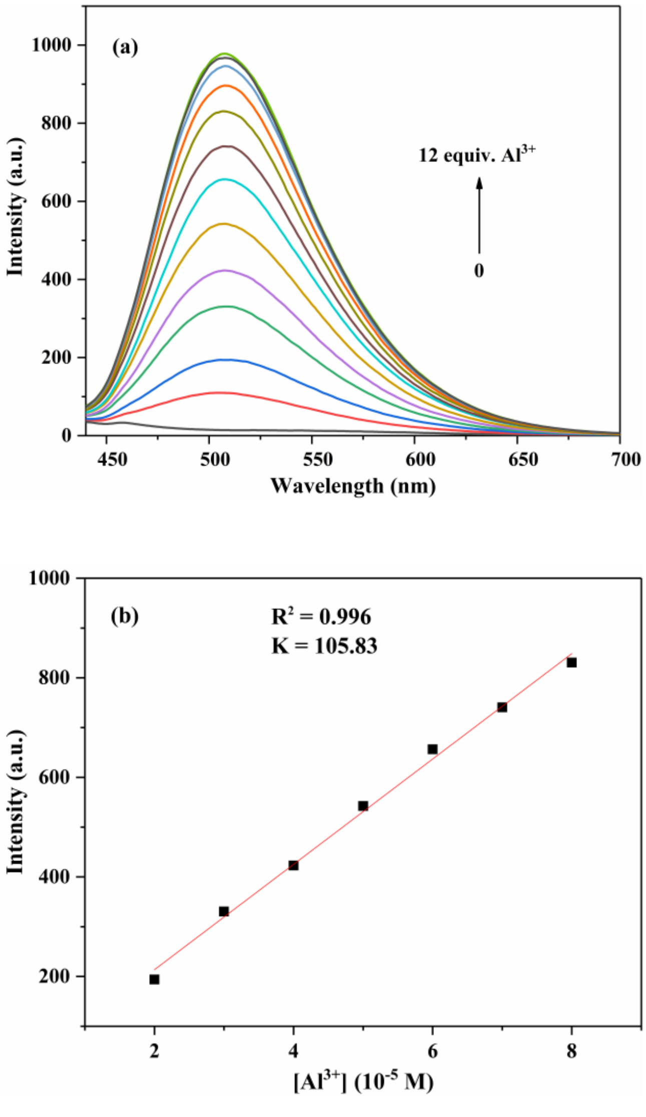

Figure S6. (a) Fluorescence emission spectra of probe **3c** (10 μM) after interaction with Al^{3+} at different concentrations (0-12.0 eq.); (b) The relationship between the maximum fluorescence intensity of probe **3c** and the concentration of Al^{3+} (2.0-8.0 eq.).

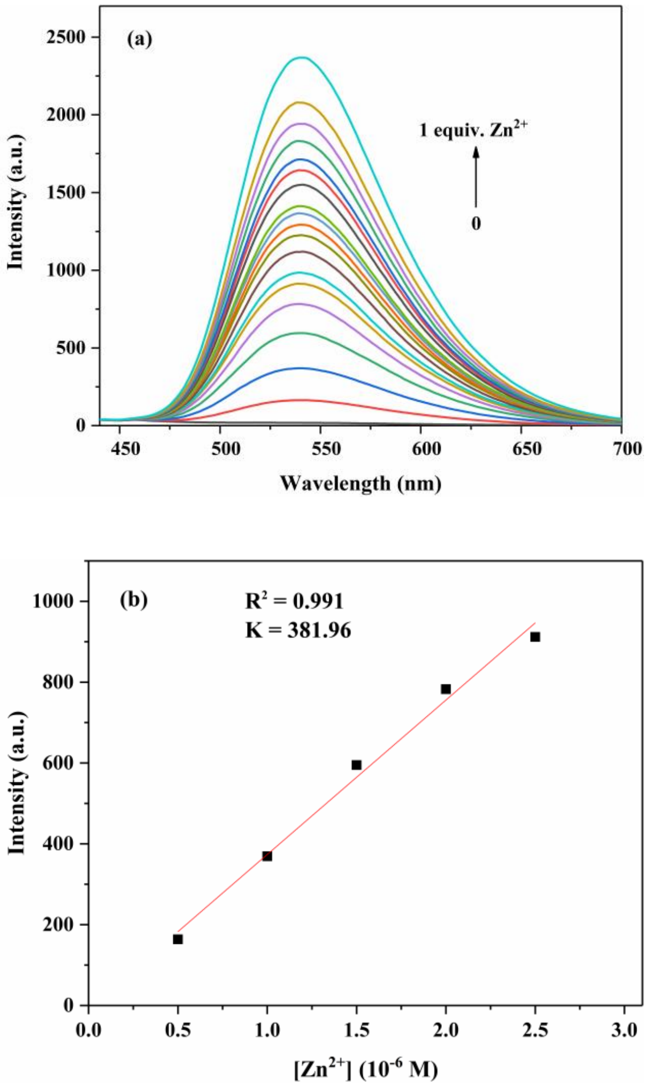

Figure S7. (a) Fluorescence emission spectra of probe **3c** (10 μM) after interaction with Zn^{2+} at different concentrations (0-1.0 eq.); (b) The relationship between the maximum fluorescence intensity of probe **3c** and the concentration of Zn^{2+} (0.05-0.25 eq.).

7. Comparison of some fluorescent probes for Al^{3+}

Table S2. Comparison of reported fluorescent probes for Al^{3+} with this work.

Probe	Response time (s)	LOD (M)	Binding constant (M^{-1})	pH range	Ref.
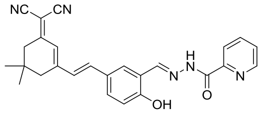	——	4.01×10^{-8}	1.83×10^{4}	5-6	[2]
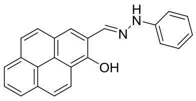	45	3.3×10^{-7}	3.08×10^{7}	2-12	[15]
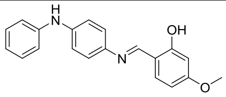	9000	1.82×10^{-6}	9.08×10^{2}	4-10	[20]
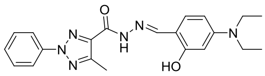	600	5.84×10^{-9}	——	——	[21]
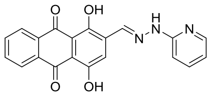	120	4.22×10^{-8}	2.9×10^{4}	3-6	[23]
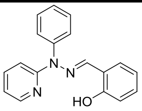	——	5.15×10^{-7}	1.08×10^{5}	6-8	[26]
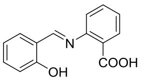	——	7.05×10^{-8}	1.67×10^{4}	6-8	[27]
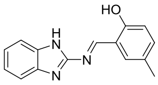 **(3a)**	90	3.14×10^{-7}	7.67×10^{3}	7.4	***This work***
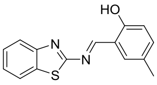 **(3b)**	80	2.81×10^{-7}	1.57×10^{4}	7.4	
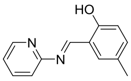 **(3c)**	80	2.86×10^{-7}	7.67×10^{3}	7-8	

8. The binding ratio and binding constant of compounds 3a and 3c interacting with Al^{3+}

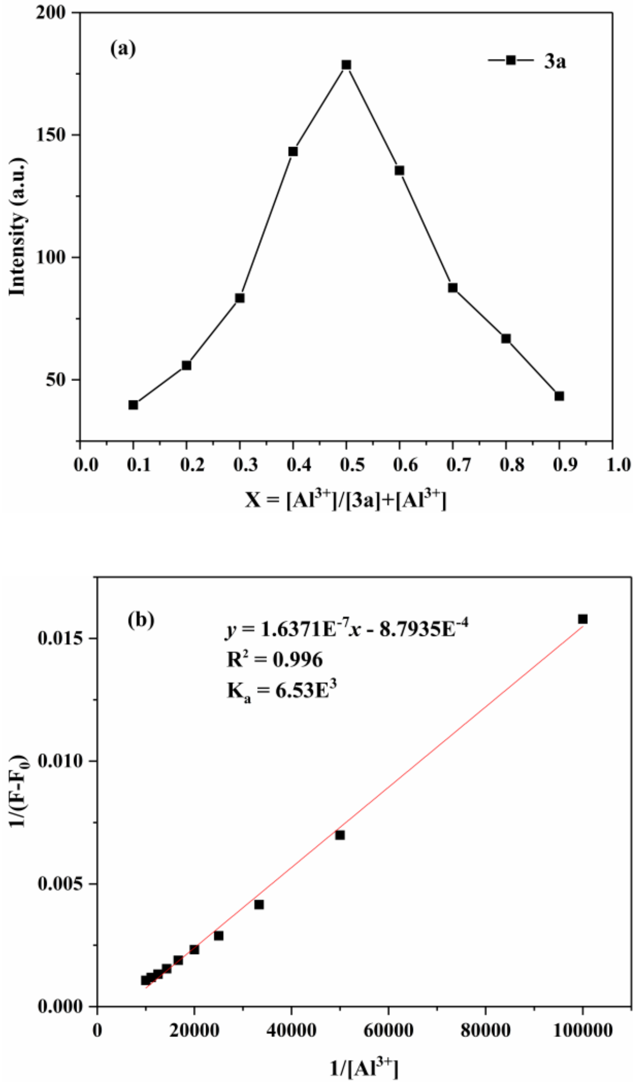

Figure S8. (a) The Job plot of **3a**-Al^{3+}; (b) The Benesi-Hildebrand plot of **3a**-Al^{3+}.

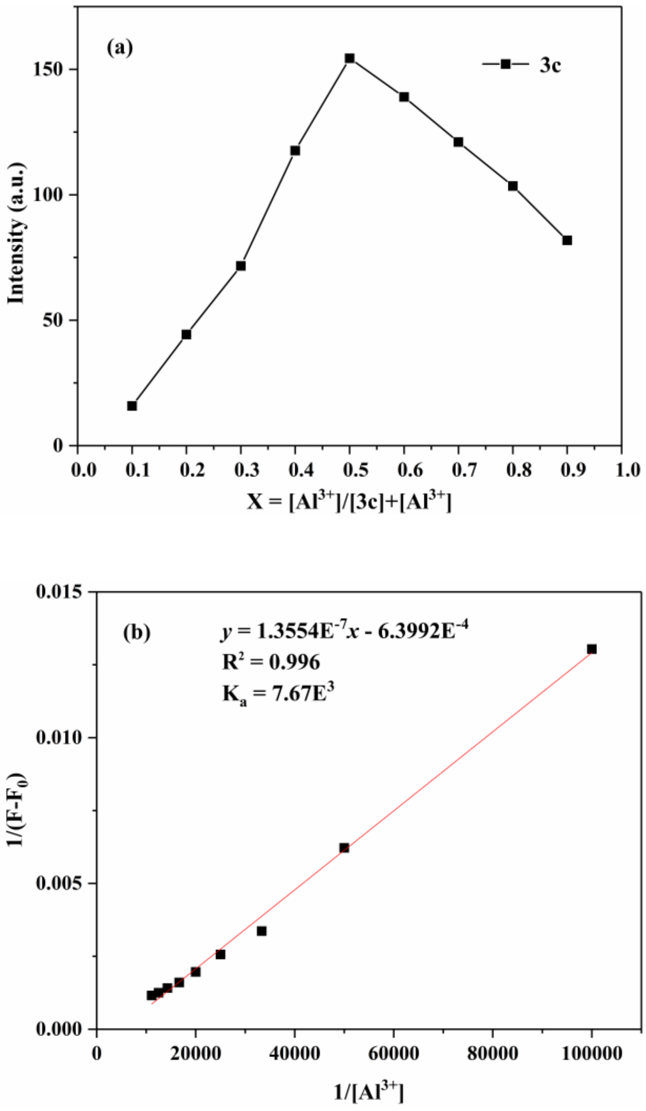

Figure S9. (a) The Job plot of **3c**-Al^{3+}; (b) The Benesi-Hildebrand plot of **3c**-Al^{3+}.

9. Study on pH tolerance of compounds 3a-3c in interaction with Al^{3+}

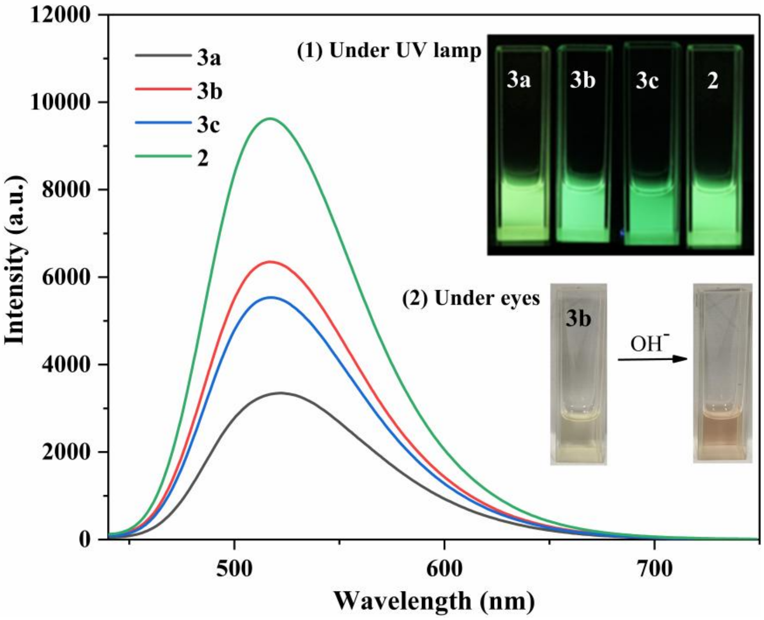

Figure S10. Fluorescence emission spectra of compounds **3a-3c** and 5-methyl salicylaldehyde, i.e., compound **2** (pH = 10, λ_{ex} = 400 nm); The inset (1) shows the fluorescence visualization image of compounds **3a-3c** and compound **2** (λ_{ex} = 365 nm); (2) shows visualization image of compound **3b** under neutral and alkaline conditions with naked eye.

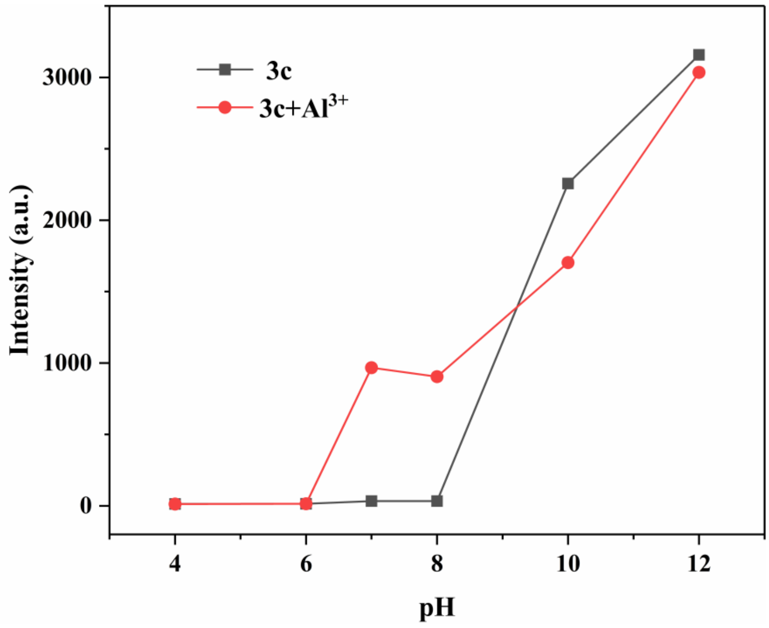

Figure S11. Different pH values of maximum fluorescence intensity before and after the interaction between compound **3c** and Al^{3+}.

10. ^{1}H NMR and FT-IR study on the interaction between compounds 3b and 3c to Al^{3+}

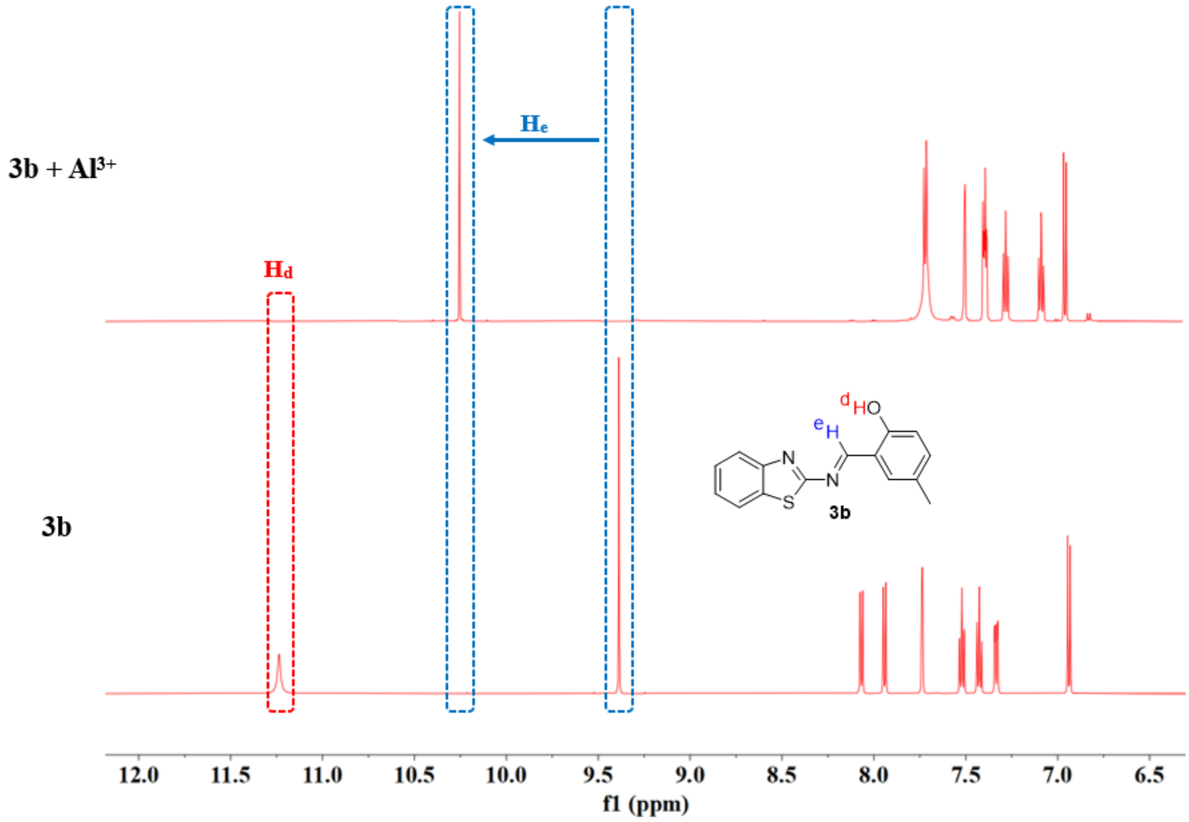

Figure S12. Hydrogen spectra of compound **3b** and **3b**+Al^{3+}.

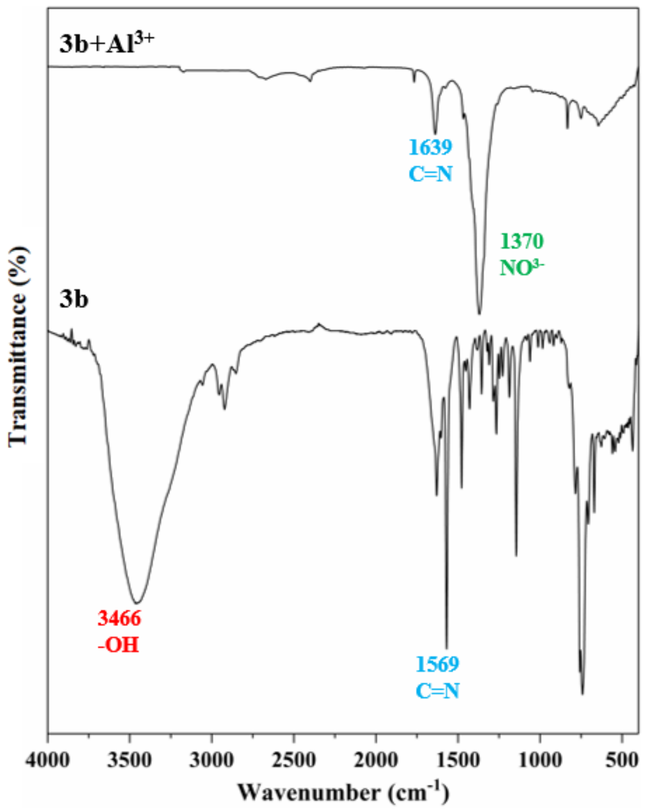

Figure S13. FT-IR spectra of compound **3b** and **3b**+Al^{3+}.

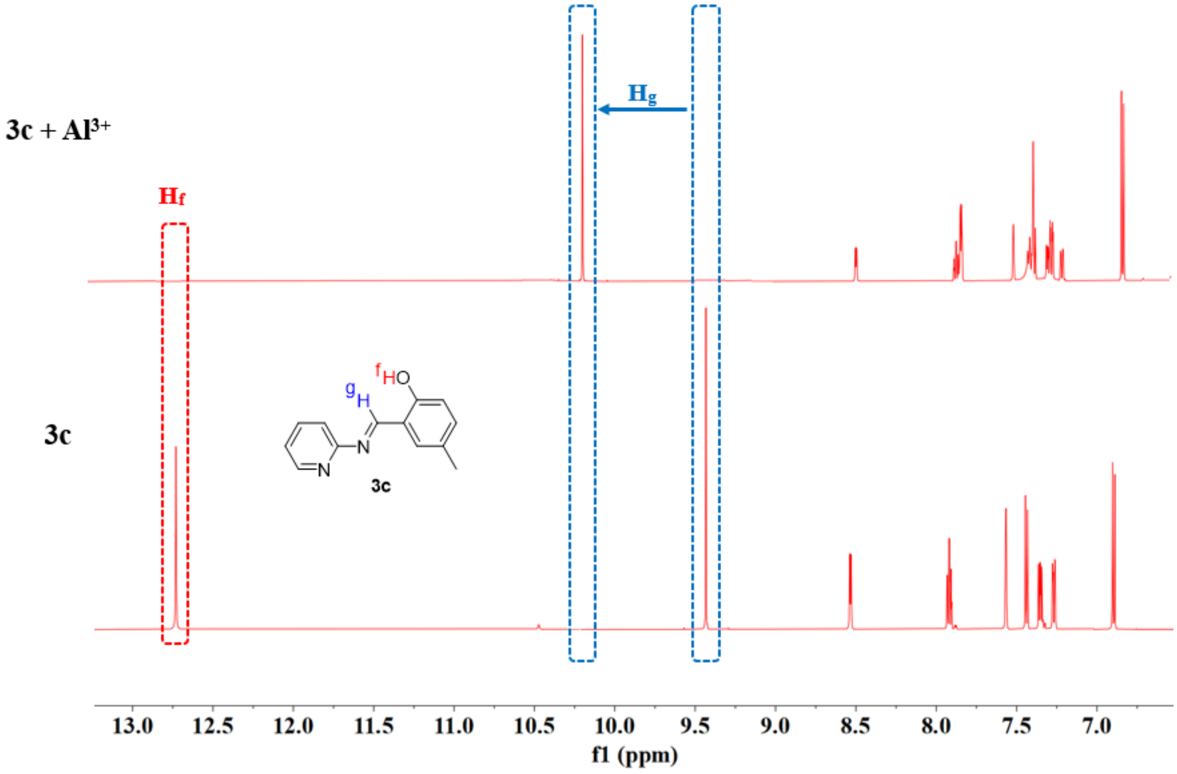

Figure S14. Hydrogen spectra of compound **3c** and **3c**+Al^{3+}.

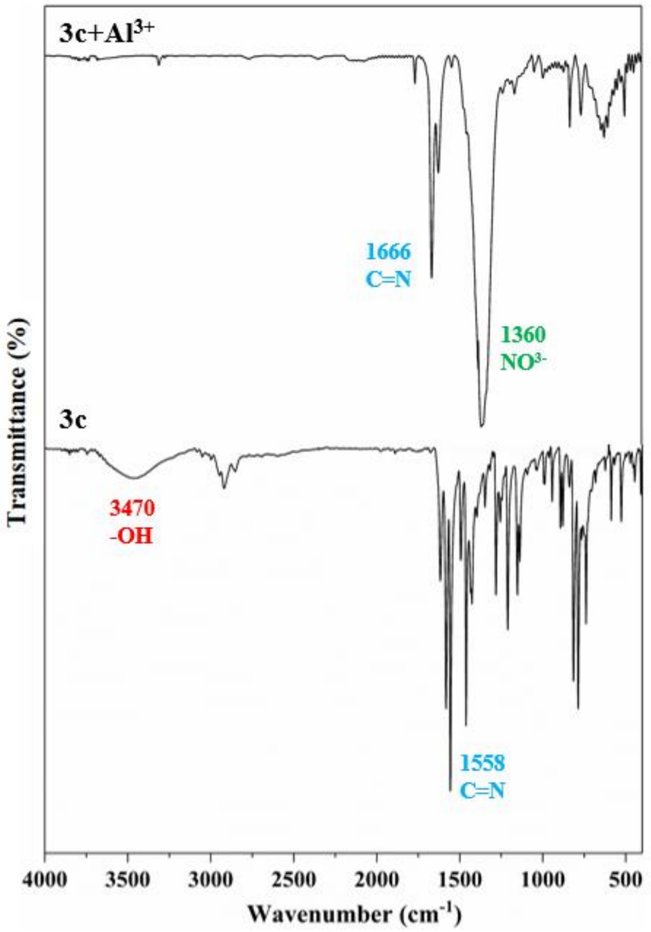

Figure S15. FT-IR spectra of compound **3c** and **3c**+Al^{3+}.

11. Application of compounds 3a and 3c in the detection of Al^{3+} in actual water and soil samples

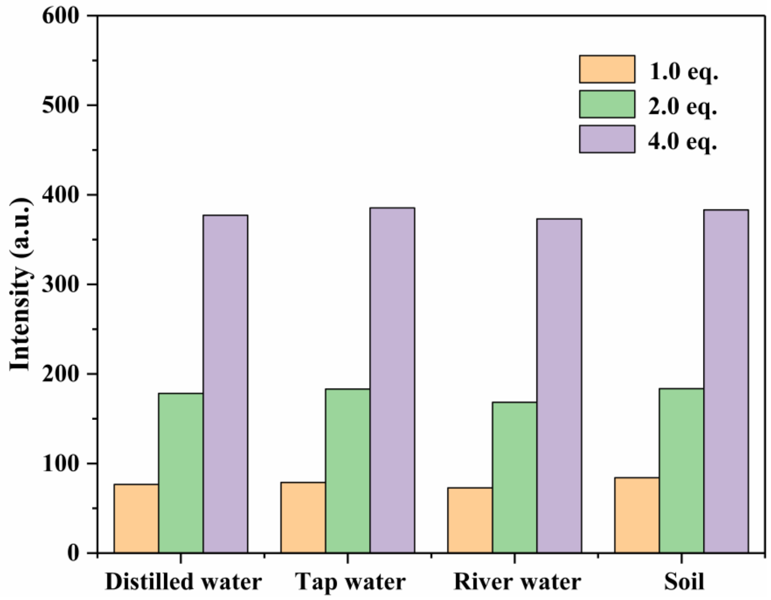

Figure S16. Relationship between maximum fluorescence intensity of compound **3a** in different water and soil samples with different concentrations of Al^{3+}.

Table S3. Determination of Al^{3+} in actual water and soil samples by compound **3a**.

**Samples**	Al^{3+} added (10^{-5} M)	Al^{3+} found (10^{-5} M)	RSD (%, n = 3)	Recovery (%)
**Distilled water**	1	1.00	1.96	100
	2	2.01	1.32	101
	4	4.00	2.45	100
**Tap water**	1	1.02	1.03	102
	2	2.06	3.16	103
	4	4.07	0.07	102
**River water**	1	0.96	0.72	96
	2	1.91	0.16	95.5
	4	3.96	1.35	99
**Soil**	1	1.07	1.50	107
	2	2.06	2.31	103
	4	4.05	2.02	101.3
**Equation**	$y = 100.21x - 23.34$			

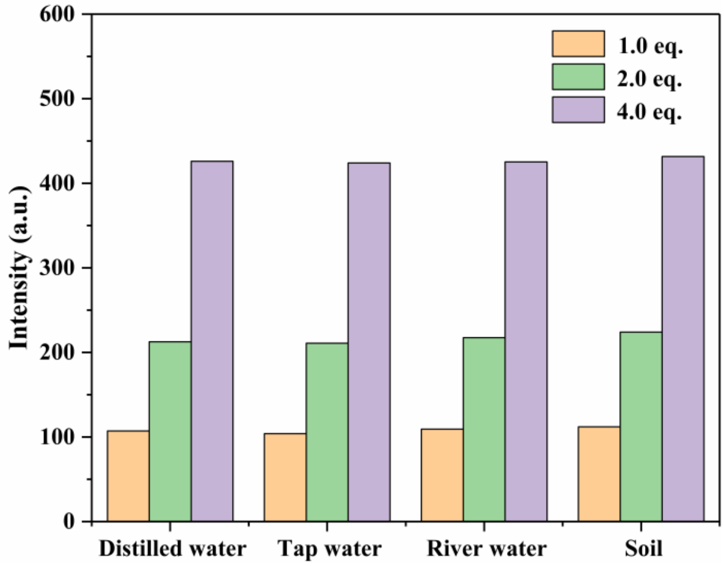

Figure S17. Relationship between maximum fluorescence intensity of compound **3c** in different water and soil samples with different concentrations of Al^{3+}.

Table S4. Determination of Al^{3+} in actual water and soil samples by compound **3c**.

**Samples**	Al^{3+} added (10^{-5} M)	Al^{3+} found (10^{-5} M)	RSD (%, n = 3)	Recovery (%)
**Distilled water**	1	0.99	0.90	99
	2	1.99	1.95	99.5
	4	4.01	0.21	100.3
**Tap water**	1	0.97	1.75	97
	2	1.97	2.67	98.5
	4	3.98	2.27	99.5
**River water**	1	1.01	1.02	105
	2	2.04	0.85	102
	4	4.00	2.45	100
**Soil**	1	1.04	0.06	101
	2	2.10	1.91	105
	4	4.06	0.16	101.5
**Equation**	$y = 105.83x + 1.96$			

12. NMR Spectra and HRMS for compounds 3a-3c

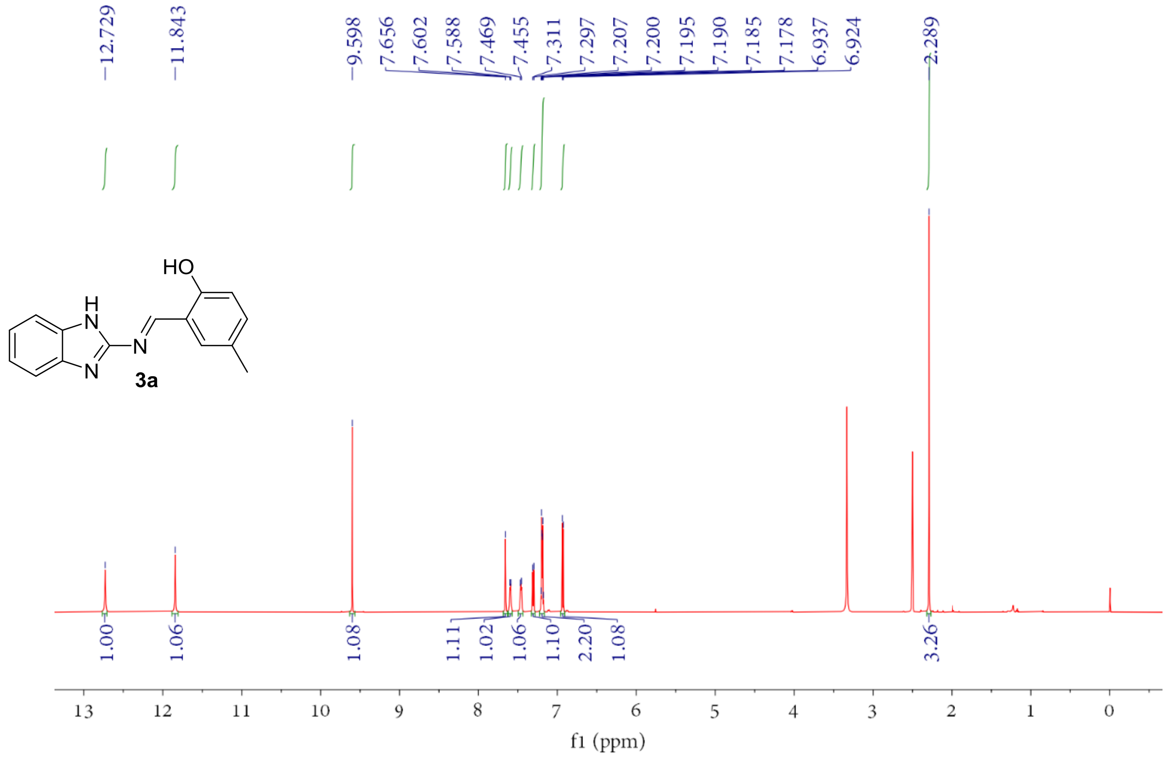

Figure S18. ^{1}H NMR spectrum of **3a** in DMSO-d_6.

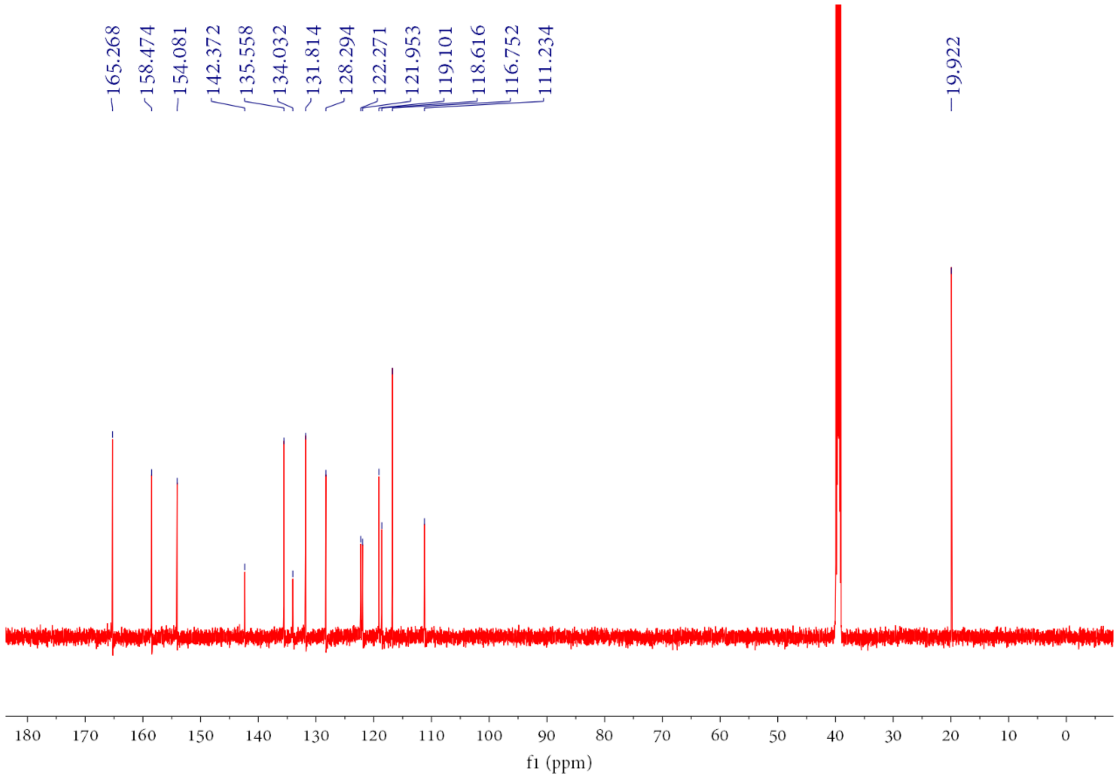

Figure S19. ^{13}C NMR spectrum of **3a** in DMSO-d_6.

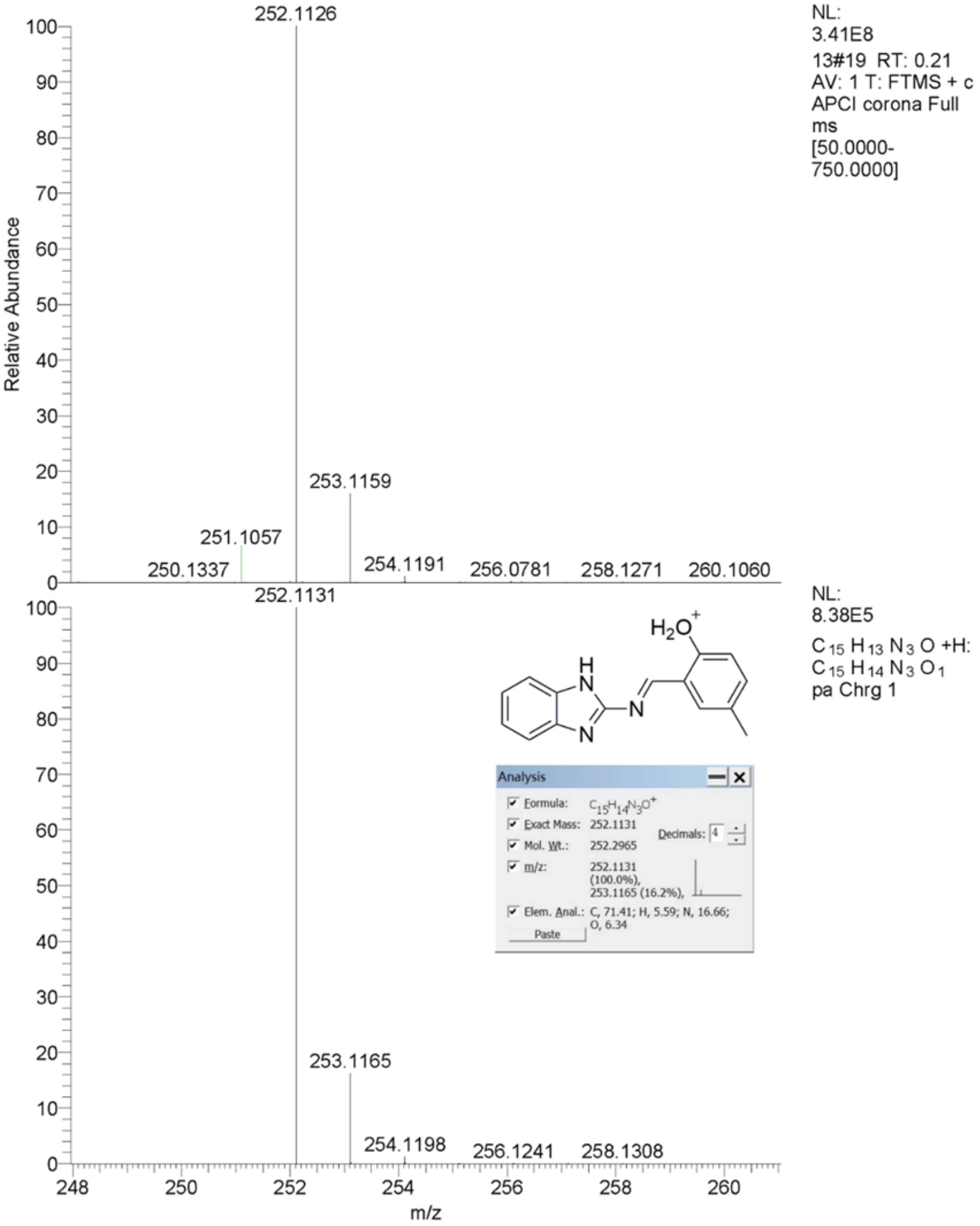

Figure S20. ESI-HRMS spectrum of **3a**.

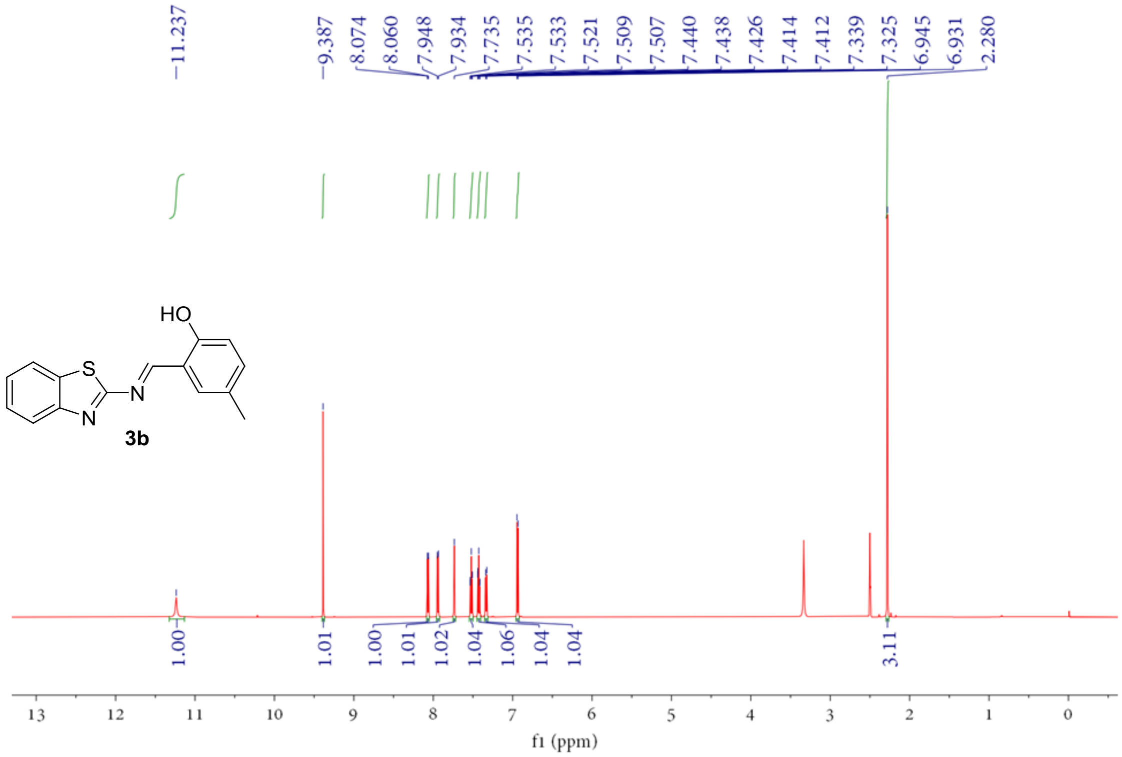

Figure S21. ^{1}H NMR spectrum of **3b** in DMSO-d_6.

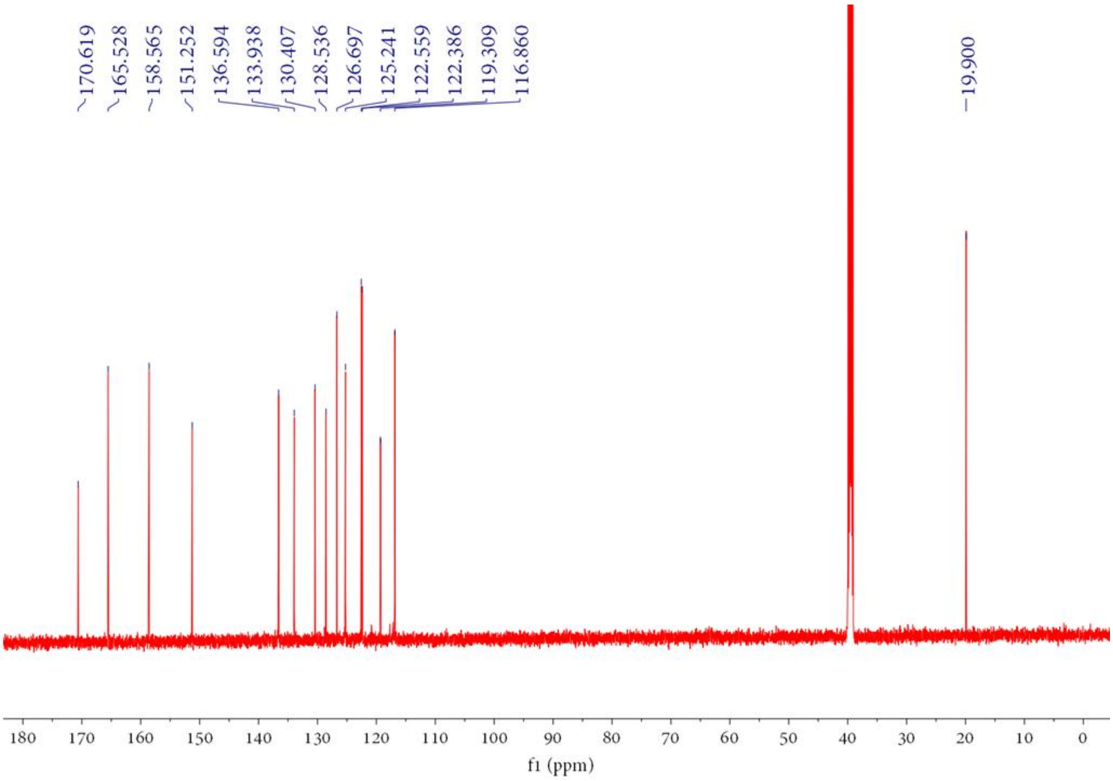

Figure S22. ^{13}C NMR spectrum of **3b** in DMSO-d_6.

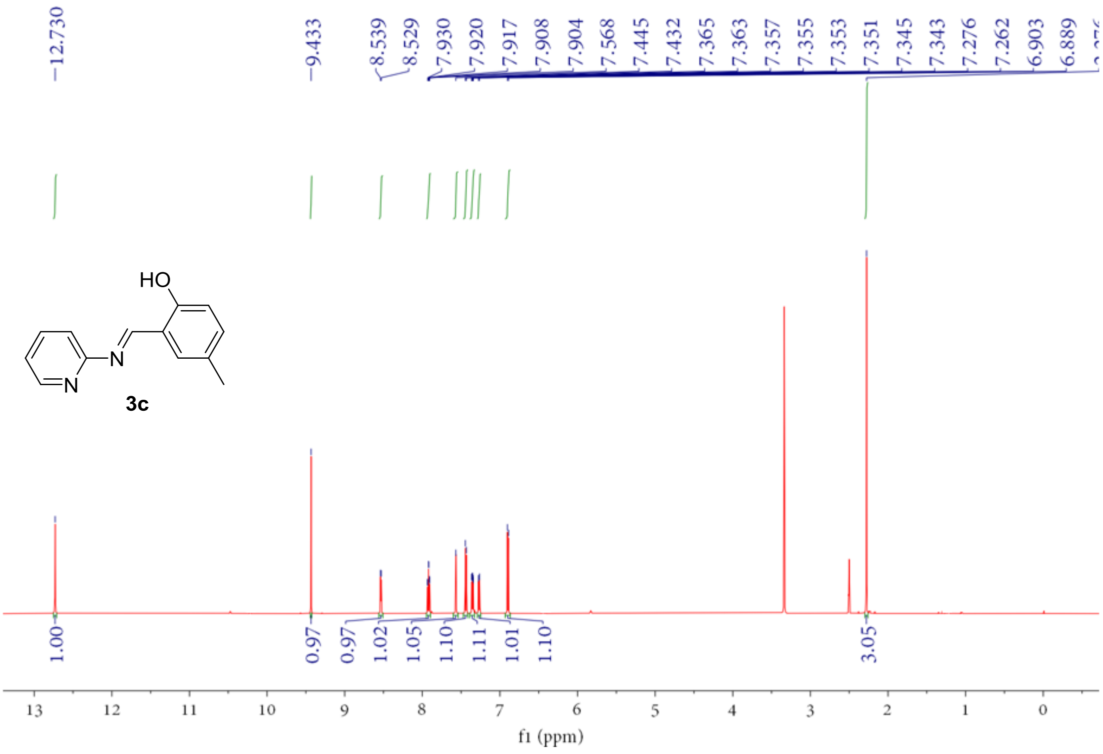

Figure S23. ^{1}H NMR spectrum of **3c** in DMSO-d_6.

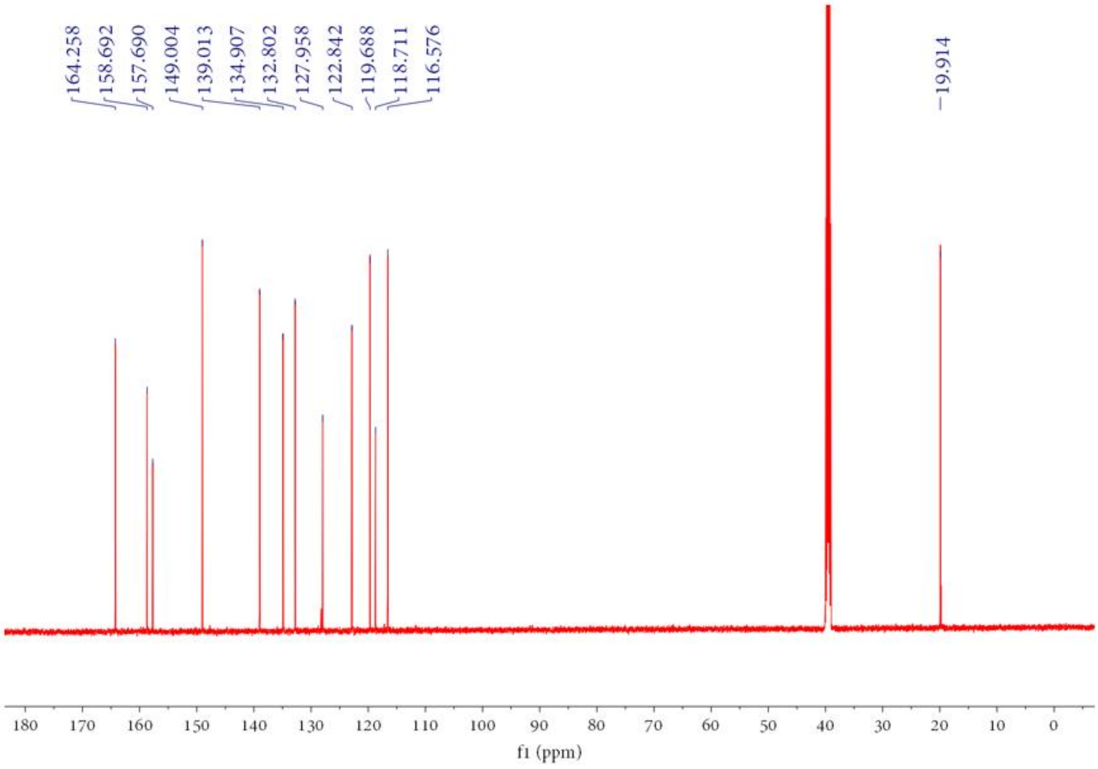

Figure S24. ^{13}C NMR spectrum of **3c** in DMSO-d_6.

References

[2] Zou, Y.-L.; Liu, Y.-T. A novel isophorone-based NIR fluorescent and colormetric probe for Al^{3+} sensing and its application for living cells and plants imaging. *Spectrochim. Acta A*, **2024**, *312*, 124040.

[15] Hu, Y.; Lu, L. M.; Wu, Y. X.; Li, Y. J.; Wang, F.; Chen, X. Q.; Fu, H. Y.; She, Y. B. Bifunctional ratiometric fluorescent membrane containing pyrene-based probe for visual monitoring and efficient removal of Al^{3+}. *Sens. Actuators, B*, **2024**, *413*, 135860.

[20] Yasar, O. G.; Elmas, S. N. K.; Aydin, D.; Arslan, F. N. Al^{3+} selective ratiometric fluorescent and colorimetric chemoprobe and its practical applications in foods, test kits and smartphone. *J. Photochem. Photobiol., A*, **2024**, *447*, 115238.

[21] Wang, S.; Liao, Y. H.; Feng, H. J.; Wu, L. Y.; He, W. Y. The studies on a fluorescent probe of Schiff base modified by 1,2,3-triazole to detecting Al^{3+} and living cell imaging. *J. Mol. Struct.*, **2024**, *1296*(1), 136730.

[23] Zhao, C.; Xu, H. M.; Meng, Y. T.; Wang, Y.; Shuang, S. M.; Dong, C. Anthraquinone-based Schiff base fluorescent probe for the sequential sensing of Al^{3+} and pyrophosphate in a near-perfect aqueous solution and bioimaging. *J. Mol. Liq.*, **2023**, *391*, 123326.

[26] Dev, K.; Singh, S.; Bhardwaj, S.; Kukreti, P.; Ramakanth, D.; Kumar, P.; Saini, S.; Roy, P.; Srivastava, V. C.; Ghosh, K.; Maji, P. K. Solvent-selective fluorescence sensing of Mg^{2+} and Al^{3+} ions by pincer-type NNO Schiff base ligand: An experimental and DFT optimized approach. *Chem. - Eur. J.*, **2024**, *30*(65), e202403256.

[27] Islam, M. S.; Hoque, A.; Baig, K. M. Y.; Sarmin, M.; Kole, G. K.; Hoda, M.; Alam, M. A. A zwitterionic probe for ratiometric fluorescent detection of aluminium(III) ion in aqueous medium and its application in bioimaging. *Spectrochim. Acta A*, **2024**, *311*, 124005.
